# Neddylation inhibition induces DNA double-strand breaks, hampering tumor growth in vivo, and promotes radiosensitivity in PAX3–FOXO1 rhabdomyosarcoma

**DOI:** 10.1038/s41420-025-02787-0

**Published:** 2025-11-03

**Authors:** Francesca Antonella Aiello, Lucrezia D’Archivio, Marika Attili, Erika Ferraro, Elisa Macrì, Riccardo Mazzocchi, Matteo Cassandri, Silvia Pomella, Valeria Tocco, Marco Pezzullo, Cristiano De Stefanis, Silvia Codenotti, Giovanni Barillari, Cinzia Marchese, Alessandro Fanzani, Francesca Megiorni, Janet Shipley, Marielle Yohe, Susanne A. Gatz, Peter J. Houghton, Giovanni Cenci, Concetta Quintarelli, Franco Locatelli, Francesco Marampon, Biagio De Angelis, Rossella Rota

**Affiliations:** 1 Department of Hematology/Oncology, Cell and Gene Therapy, Bambino Gesù Children’s Hospital, IRCCS, Rome, Italy; 2 Department of Experimental Medicine, “Sapienza” University of Rome, Rome, Italy; 3 Department of Clinical Sciences and Translational Medicine, University of Rome Tor Vergata, Rome, Italy; 4 Core Facilities Research Laboratories, Bambino Gesù Children’s Hospital, IRCCS, Rome, Italy; 5 Department of Molecular and Translational Medicine, University of Brescia, Brescia, Italy; 6 Sarcoma Molecular Pathology, Divisions of Molecular Pathology, The Institute of Cancer Research, London, UK; 7 Laboratory of Cell and Developmental Signaling, National Cancer Institute, NIH, Frederick, MD USA; 8 Institute of Cancer and Genomic Sciences, University of Birmingham, Birmingham, West Midlands UK; 9 Greehey Children’s Cancer Research Institute (GCCRI), UT Health Science Center, San Antonio, TX USA; 10 Department of Biology and Biotechnologies “C. Darwin”, “Sapienza” University of Rome, Rome, Italy; 11 Department of Clinical Medicine and Surgery, University of Naples Federico II, Naples, Italy; 12 Department of Life Sciences and Public Health, Catholic University of the Sacred Heart, Rome, Italy; 13 Department of Radiotherapy, Policlinico Umberto I, “Sapienza” University of Rome, Rome, Italy

**Keywords:** Paediatric cancer, DNA damage and repair, Sarcoma

## Abstract

Rhabdomyosarcoma (RMS) is an aggressive soft tissue sarcoma with myogenic features affecting children and adolescents. The high-risk fusion-positive RMS subtype (FP-RMS), driven by the oncogenic chimeric transcription factor PAX3–FOXO1, shows 5-year overall survival not exceeding 30%. Here, we examine the impact of neddylation inhibition, a post-translational modification in which the NEDD8 peptide is conjugated to proteins, on the tumorigenic properties of FP-RMS. Here, we report that the *NAE1* and *UBA3* genes encoding the two subunits of the NEDD8-activating enzyme (NAE) heterodimer are upregulated in FP-RMS patients compared to healthy skeletal muscle tissues and highly expressed in RMS among several tumor types. Furthermore, DepMap analyses showed that FP-RMS cell lines are among the most sensitive to both *NAE1* and *UBA3* CRISPR-mediated knockout as well as to NAE pharmacological inhibition with MLN4924 compared to other cancer cell lines. In agreement, FP-RMS cells treated in vitro with MLN4924 (Pevonedistat) exhibited cell proliferation decrease, G2/M cell cycle arrest, senescence, and caspase- and PARP1-dependent apoptosis. These phenotypes were associated with increased γH2AX nuclear foci and protein levels, DNA double-strand breaks (DSB), and reduced RAD51 levels. *NAE1* and *UBA3* individual silencing mirrors the major effects of MLN4924. In addition, MLN4924 also prevented FP-RMS tumor growth in vivo. Combining MLN4924 with irradiation enhanced apoptosis and the inhibition of colony formation, cell cycle progression, and anchorage-independent and tumor spheroids growth compared to single treatments. Molecularly, MLN4924 amplified the irradiation-induced DNA damage by increasing γH2AX and DSBs, while reducing RAD51 expression and DNA-PKcs activation, both of which are involved in DNA repair. Collectively, our results suggest that the neddylation pathway is deregulated in FP-RMS, representing a potential therapeutic target. Therefore, MLN4924 could be considered as an anti-tumorigenic compound and a novel radiosensitizer in FP-RMS.

## Introduction

Pediatric rhabdomyosarcoma (RMS) is the most common soft tissue sarcoma of childhood, accounting for about 8% of all pediatric solid tumors [1]. RMS cells express the lineage-specifying myogenic transcription factors (TF) MYOD and MYOG but have lost the ability to differentiate into skeletal muscle and proliferate indefinitely. The two main RMS molecular subtypes are characterized by the expression of the fusion TF PAX3–FOXO1 (fusion-positive, FP-RMS), the malignancy driver, or are devoid of gene fusions and frequently harbor mutations in the RAS pathway (fusion-negative, FN-RMS) [2, 3]. PAX3–FOXO1 FP-RMS are at ultra-high risk, showing a 5-year overall survival rate less than 30%, possibly related to intrinsic chemo- and radio-resistance [4, 5]. Thus, novel therapeutic strategies to enhance the survival of PAX3–FOXO1 RMS patients are urgently needed.

Neddylation is an enzymatic post-translational modification that covalently conjugates the ubiquitin-like protein NEDD8 to substrates to regulate many aspects of cell homeostasis [6]. The NEDD8-activating enzyme (NAE), a heterodimer formed by the regulatory NAE1 and the enzymatic UBA3 subunits, is responsible for the activation of NEDD8, preceding its conjugation to target substrates [7]. Neddylation of the scaffold proteins cullins is essential for the activation of the Cullin RING ligases (CRL) complexes, the largest E3-ligase group, which target proteins for ubiquitylation and proteasomal degradation [8].

Importantly, neddylation of Cullin 1 (CUL1) activates the CRL-1 E3-ligase, promoting the degradation of the cyclin-dependent kinase (CDK) inhibitors p21 and p27, through the binding with the oncogenic CRL-1 F-box protein S-phase kinase-associated protein-2, SKP2, leading to cell cycle progression and tumorigenesis [9–11]. Moreover, the neddylation pathway has been shown to be aberrantly activated in cancer due to the overexpression of different components, representing a promising target for therapy [6, 12, 13]. To date, the NAE inhibitor MLN4924 (Pevonedistat) is being evaluated in Phase I and II clinical studies in monotherapy or in combination in adult cancers [14–17]. Moreover, it is being tested in Phase I trials in combination with Irinotecan and Temozolomide in young patients with relapsed or refractory solid tumors [18].

In this study, we show that both *NAE1* and *UBA3* are overexpressed in FP-RMS patients compared to healthy muscle tissues and several other tumor types. Moreover, FP-RMS cell lines were among the most sensitive cancer cell lines to both *NAE1* and *UBA3* genetic depletion as well as NAE pharmacological inhibition with MLN4924 compared to the other cell lines. We then demonstrate that, in two FP-RMS cell lines, MLN4924 treatment led to a reduction in the proliferation rate, G2/M cell cycle arrest, cell senescence, and caspase 3/7- and PARP1-dependent apoptotic cell death. These functional effects were associated with upregulation of p21 and p27 protein levels, as well as increased expression of the DNA damage marker γH2AX and DNA double-strand breaks (DSB) formation. Concomitantly, the protein levels of the DNA damage repair factor RAD51 were downregulated. NAE1 and UBA3 individual silencing phenocopied the effects of the drug. MLN4924 treatment also hampered tumor growth in vivo in the two FP-RMS models. Moreover, MLN4924 radiosensitized the intrinsically radioresistant FP-RMS cells in vitro by enhancing the irradiation-induced (i) decrease of cell growth, colony formation ability, and anchorage-independent growth, and (ii) increase of DSBs and by reducing DNA-PKcs phosphorylation/activation compared to single treatments.

In summary, our results demonstrate that inhibition of neddylation could be used to hinder the tumorigenic abilities of FP-RMS and boost the response to irradiation.

## Results

### The NEDD8-activating enzyme (NAE) regulatory and catalytic components, *NAE1* and *UBA3*, are upregulated in RMS, and the FP-RMS cells are dependent on their functions

**Fig. 1:**
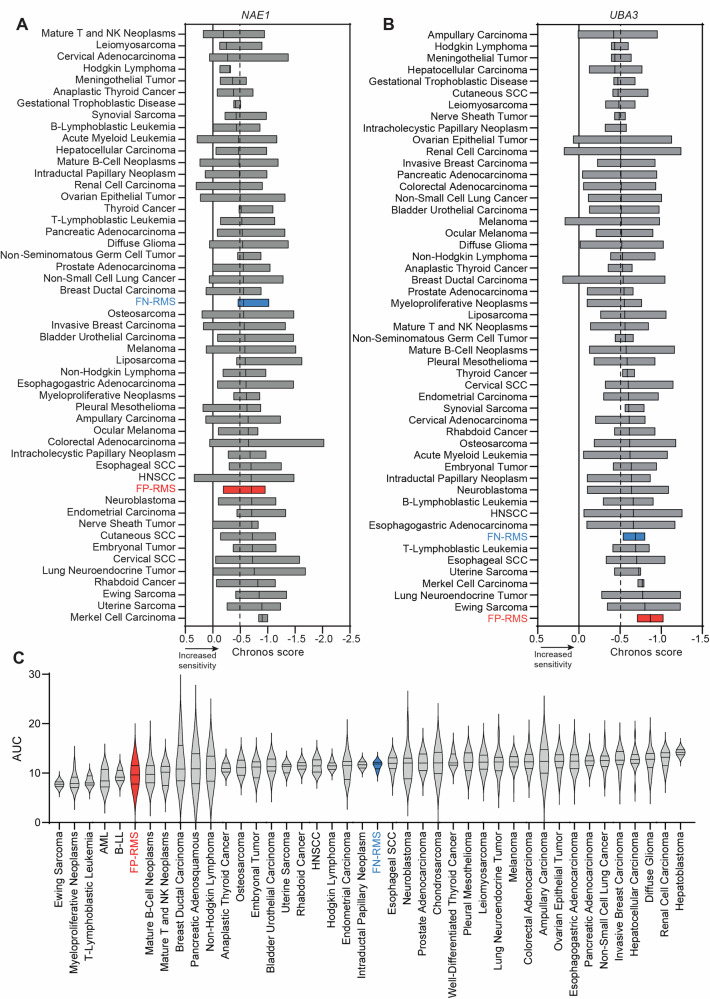
The NEDD8-activating enzyme (NAE1) is upregulated in RMS patients, and FP-RMS cells are dependent on its functions. **A**, **B** Floating bars plot for *NAE1* and *UBA3* CRISPR depletion across several tumor types from Achilles project (24Q2 DepMap, https://depmap.org/portal/achilles/). Floating bars depict minimum and maximum Chronos score values, and the black line in the bar shows the median. The full line is set at a score of 0 (not essential gene), and the dashed line is set at a score of −0.5 (essential gene). **C** Violin plot depicts MLN4924 drug response in different cell lines from drug sensitivity AUC (CTD^2^) (24Q2 DepMap, https://depmap.org/portal/). AUC area under the curve.

To investigate the potential impact of neddylation in RMS, we analyzed the level of gene expression of *NAE1* and *UBA3*, encoding for the regulatory and catalytic subunit of the heterodimeric NAE complex, respectively. Analyses of two RMS patient cohorts show that both genes were upregulated in RMS patients compared to normal skeletal muscle tissues, regardless of the fusion status, with the exception of the expression of *UBA3*, which was higher in FP-RMS *vs* FN-RMS patients in one of the two datasets analyzed, in line with the heterogeneity of the tumor (Supplementary Fig. S1A, B). Moreover, RMS are among the tumors with the highest expression of the two genes across different adult and pediatric tumors (Supplementary Fig. S1C, D). Therefore, we explored the effects of individual *NAE1* and *UBA3* CRISPR/Cas9 knockout (KO) on cell survival analyzing the data from Achilles Project (https://depmap.org/portal/achilles) where the lower the Chronos score, the more a cell line is dependent on gene expression. The analyses showed that FP-RMS cell lines were the most dependent on *UBA3* expression and one of the most dependent cell lines on *NAE1* expression compared to different adult and pediatric tumor cell lines and to FN-RMS as well (Chronos score −0.70 and −0.56 for *NAE1* and −0.87 and −0.69 for *UBA3* in FP-RMS and FN-RMS cells, respectively, with Chronos score < −0.5 is considered a significant dependency) (Fig. 1A, B). The vulnerability of FP-RMS cells on the functions of NAE was further confirmed by the analysis of the CTD^2^ drug sensitivity dataset for MLN4924 (https://depmap.org/portal/achilles). Figure 1C shows that FP-RMS cells are among the most sensitive tumor cell lines exhibiting the lowest values of the area under the curve (AUC; the lower the AUC, the higher drug sensitivity) across all the tumor cell lines analyzed. Altogether, these data show that FP-RMS cells express high levels of *NAE1* and *UBA3* and are dependent on NAE functions.

### MLN4924 impairs cell proliferation and survival in FP-RMS cells

**Fig. 2:**
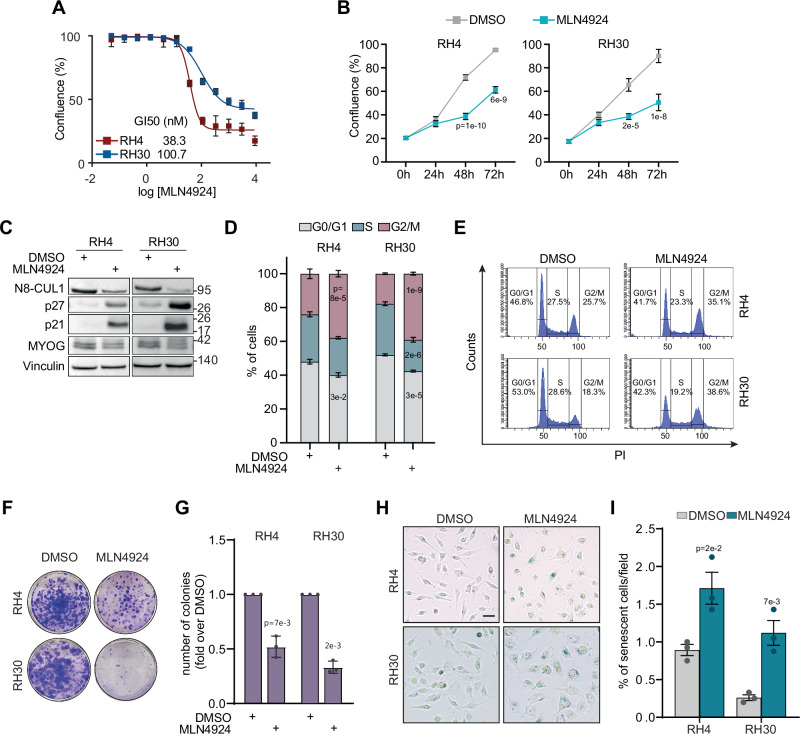
MLN4924 impairs cell proliferation and survival in FP-RMS cells. **A** Dose–response curve of RH4 and RH30 cell lines treated with increasing concentration of MLN4924. Values of GI_50_ (Growth Inhibition 50%) are reported in the figure. Graph depicts the mean ± SEM (*n* = 3 independent experiments). **B** Growth-curve analysis of RH4 and RH30 cell lines treated with either vehicle (DMSO) or MLN4924 GI_50_ at 0, 24, 48, and 72 h. Graph represents the mean ± SD (*n* = 3 independent experiments). Two-way ANOVA. Exact *p*-values are reported in the figure. **C** Representative western blot (*n* = 3 independent experiments) of the indicated proteins on RH4 and RH30 cells treated for 48 h with either vehicle (DMSO) or MLN4924 GI_50_. Vinculin is the loading control. **D** Histograms depict the percentage of RH4 and RH30 cells treated as in (**C**) in G0/G1, S, and G2/M phases. Graph represents the mean ± SEM (*n* = 3 independent experiments). Two-way ANOVA. Exact *p*-values are reported in the figure. **E** Representative diagrams of cell cycle flow cytometry analysis on RH4 and RH30 cells treated as in (**C**). **F** Representative images of RH4 and RH30 colonies treated as in (**C**). Colonies were stained with crystal violet 12 days post-cell seeding. **G** Histograms depict the number of colonies of RH4 and RH30 cells treated as in (**C**) calculated as fold increase over vehicle (DMSO). Graph represents the mean ± SD (*n* = 3 independent experiments). Two-tailed *t*-test. Exact *p*-values are reported in the figure. **H** Representative pictures of β-Galactosidase staining of RH4 and RH30 cells treated as in (**C**). Scale bar = 100 μm. **I** Histograms depict the percentage of senescent cells per field. Bars represent the mean ± SEM (*n* = 3 independent experiments). Two-tailed *t*-test. Exact *p*-values are reported in the figure.

Given the dependency of FP-RMS cells on *NAE1* and *UBA3*, we decided to functionally explore the effects of the pharmacological inhibition of NEDD8 neddylation. We show here that a GI_50_ (growth inhibition) dose of MLN4924 slowed down cell proliferation in two FP-RMS cells, RH4 and RH30, both expressing *PAX3-FOXO1*, decreasing cell confluence by approximately 40% 72 h post-treatment in both cell lines compared to treatment with vehicle (DMSO) (Fig. 2A, B). The inhibitory effects on NAE functions by MLN4924 were testified by the reduction of the protein levels of neddylated CUL1 (NEDD8(N8)-CUL1) *vs* vehicle (Fig. 2C). As a result of CUL1 neddylation blockade and in line with the decrease of cell proliferation, protein levels of p21 and p27 were upregulated in both cell lines upon 48 h of MLN4924 treatment [9, 11, 19, 20] (Fig. 2C). Accordingly, drug treatment resulted in cell cycle arrest leading to significant accumulation of cells in the G2/M phase of approximately 1.6- and 2.2-fold for RH4 and RH30 cells, respectively, compared to vehicle-treated cells (Fig. 2D, E).

The clonogenic ability was also reduced by about 50% and 70% in MLN4924- *vs* vehicle-treated RH4 and RH30 cells, respectively (Fig. 2F, G). Finally, FP-RMS cells also acquired a senescent phenotype 48 h after MLN4924 treatment, as shown by approximately 1.9- and 4.3-fold increase in β-galactosidase-positive cells compared to vehicle-treated RH4 and RH30 cells, respectively (Fig. 2H, I). Similar results were seen after genetic depletion using oligo siRNAs of *NAE1* and *UBA3* (Supplementary Fig. S2). Knockdown of both genes affected the ability of FP-RMS cells to survive, promoted p21 and p27 upregulation and MYOG reduction, and induced senescence. However, cell accumulation in G2/M phase was only seen after NAE1 silencing, suggesting that NAE1 is needed for cell cycle progression (Supplementary Fig. S2A–F). Collectively, these results demonstrate that neddylation inhibition with MLN4924 promotes G2/M cell cycle arrest and senescence and hinders colony formation ability in FP-RMS cells.

### MLN4924 promotes caspase-dependent apoptosis and DSBs in PAX3–FOXO1 RMS cells

**Fig. 3:**
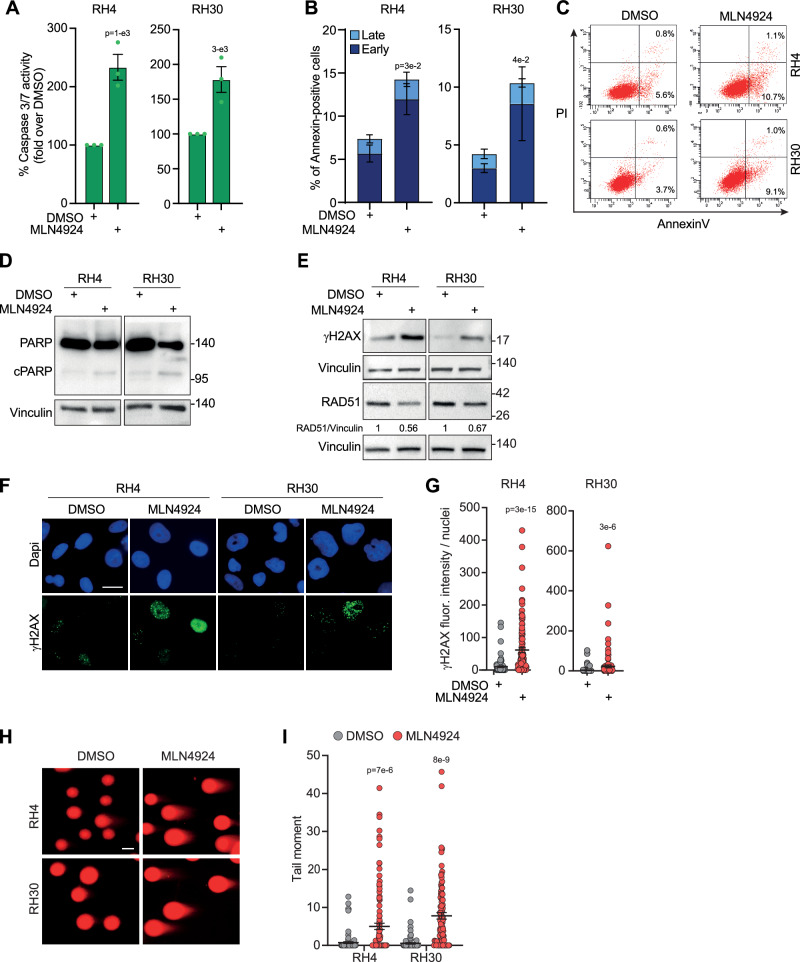
MLN4924 promotes caspase-dependent apoptosis and DSBs in FP-RMS cells. **A** Histograms depict the percentage of Caspase 3/7 activity of RH4 and RH30 cells treated for 48 h with either vehicle (DMSO) or MLN4924 GI_50_ calculated as fold increase over vehicle (DMSO, 100%). Graph represents the mean ± SEM (*n* = 3 independent experiments). Two-tailed *t*-test. Exact *p*-values are reported in the figure. **B** Histograms depict the percentage of Annexin-V positive/PI single- and double-positive RH4 and RH30 cells treated as in (**A**). Graph represents the mean ± SEM (*n* = 3 independent experiments). Two-tailed *t*-test. Exact *p*-values are reported in the figure. **C** Representative plots of flow cytometry analysis of Annexin V/PI staining of RH4 and RH30 cells treated as in (**A**). **D**, **E** Representative western blot (*n* = 3 independent experiments) of the indicated proteins on RH4 and RH30 cells treated as in (**A**). Vinculin is the loading control. **F** Representative immunofluorescence images of γH2AX (green) on RH4 and RH30 cells treated as in (**A**). Dapi (blue) was the nuclear counterstain. Images were acquired with a fluorescence microscope equipped with a 60× oil immersion objective. Scale bar = 25 μm. **G** Scatter dot plots depict the intensity of γH2AX staining per nucleus on RH4 and RH30 cells treated as in (**A**). The graph represents the mean ± SEM. Kruskal-Wallis test. Exact *p*-values are reported in the figure. **H**. Representative immunofluorescence images of the neutral Comet assay on RH4 and RH30 cells treated as in (**A**). Images were acquired with a fluorescence microscope equipped with a 40× oil immersion objective. Scale bar = 50 μm. **I** Scatter dot plots represent the mean of the tail moment ± SEM. Mann–Whitney test. Exact *p*-values are reported in the figure.

Next, to clarify the mechanism underlying the hindered FP-RMS cells’ growth, we evaluated whether MLN4924 was able to induce apoptotic cell death. We show that the activity of caspase 3/7 was approximately doubled in RH4 and RH30 cells 48 h after drug treatment compared to vehicle (Fig. 3A). This was in line with an about 2-fold increase of Annexin V-positive apoptotic cells (Fig. 3B, C) and induction of PARP cleavage by the drug compared to vehicle (Fig. 3D). Since MLN4924 treatment has been recently reported to induce DNA damage [21] we investigated whether the apoptotic response was related, at least in part, to an accumulation of DNA damage in our tumor context. Figure 3E shows a marked increment of the protein levels of the DNA damage histone marker γH2AX after 48 h of MLN4924 treatment compared to vehicle in both RH4 and RH30 cells. This was consistent with the significant increase in nuclear γH2AX, which is known to be recruited to the DNA-damaged sites [22], demonstrated by about 6- and 7-fold elevation of the nuclear fluorescence intensity of γH2AX foci in RH4 and RH30 cells, respectively (Fig. 3F, G). Interestingly, this phenomenon was associated with the concomitant decrease of the protein levels of RAD51, a key factor for DNA repair [23] (Fig. 3E). We, thus, explored the ability of the neddylation inhibitor to induce DSBs using the neutral Comet assay, a recognized method based on the detection, at the single cell level, of fragments of DNA strands that migrate from the nucleus forming a tail-like comet structure (tail moment) [24]. Forty-eight hours of MLN4924 treatment resulted in approximately 15- and 13-fold increase of tail moment in RH4 and RH30 cells, respectively, indicating persistent accumulation of DSBs in treated cells compared to vehicle-treated ones (Fig. 3H, I). Notably, the increase of γH2AX and cleaved PARP, together with the concomitant reduction of RAD51, supported the induction of apoptosis after *NAE1* silencing (Supplementary Fig. S2C, G). Interestingly, *UBA3* knockdown did not increase PARP cleavage compared to control cells and induced significant Annexin V positivity (apoptotic cells) only in RH4 cells. Altogether, these data suggest that inhibiting neddylation with MLN4924 induces caspase-dependent cell death and double-strand DNA damage in FP-RMS cells.

### Neddylation inhibition by MLN4924 prevents FP-RMS tumor growth in vivo

**Fig. 4:**
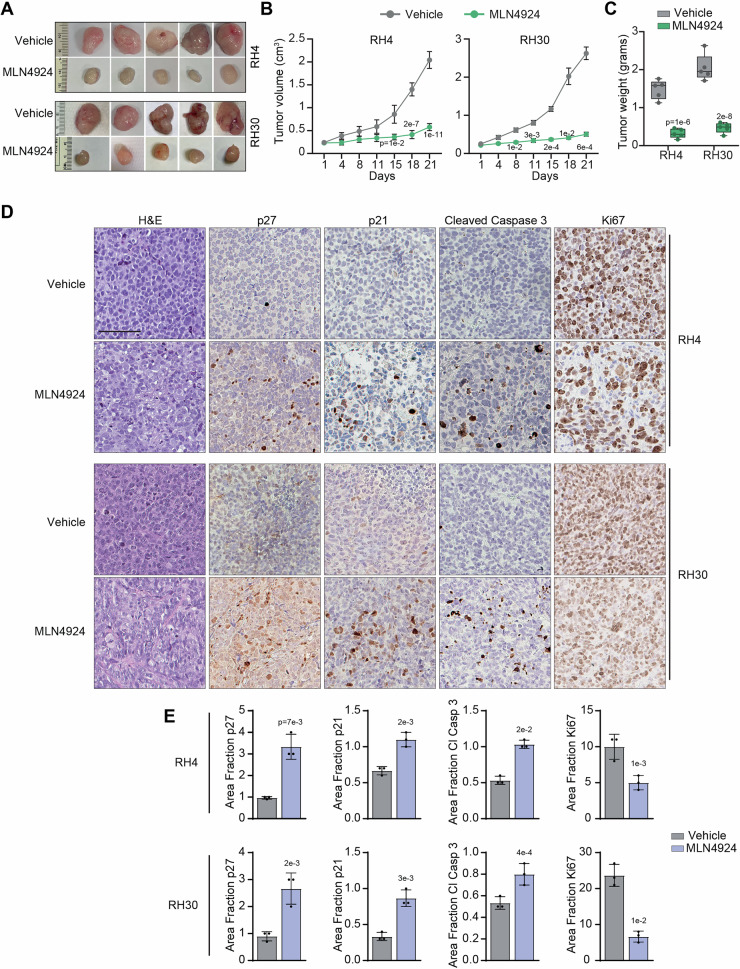
MLN4924 prevents PAX3–FOXO1 RMS tumor growth in vivo. **A** Images of RH4 and RH30 xenografted tumors explanted from mice treated for 3 weeks with either vehicle or MLN4924 50 mg/kg. **B** Volume of RH4 and RH30 xenografted tumors treated as in (**A**) (vehicle *n* = 6, MLN4924 *n* = 6). Data presented the mean ± SEM. Two-way ANOVA. Exact *p*-values are reported in the figure. **C** Box plot depicts the weight of RH4 and RH30 xenografted tumors explanted from mice treated as in (**A**). Box plots represent 25th to 75th quartiles, the black bar depicts the median, and whiskers go to the lower quartile and then to the upper quartile. Two-way ANOVA. Exact *p*-values are reported in the figure. **D** Representative images of H&E, p27, p21, Cleaved Caspase, and Ki67 immunohistochemistry of tumor sections from RH4 and RH30 xenografts explanted from mice treated as in (**A**). Scale Bars = 100 μm. **E** Histograms depict the quantification of the Area fraction of the immunohistochemistry staining reported in (**D**). Graph represents the mean ± SD (*n* = 3 independent tumor sections). Two-tailed *t*-test. Exact *p*-values are reported in the figure.

To investigate the therapeutic potential of our findings, we evaluated the response of FP-RMS cells to MLN4924 in vivo. Thus, RH4 and RH30 cells were subcutaneously inoculated in immunodeficient mice that, when the tumors reached about 0.200 cm^3^, were treated with 50 mg/kg of MLN4924 6 days a week for 3 weeks [9, 11]. As shown in Fig. 4, MLN4924 treatment markedly inhibited the tumor growth of both cell lines compared to the control vehicle. Indeed, the increment of tumor mass at the end of the experiment over day 1 was approximately 2.5- and 2.3-fold in MLN4924 *vs* 8.5- and 10.4-fold in control vehicle for RH4 and RH30 xenografts, respectively (Fig. 4A, B). The tumor weight was also highly reduced in MLN4924-treated mice compared to vehicle-treated ones (Fig. 4C).

In agreement with the growth inhibition, immunohistochemical analysis on tumor sections from mice treated with the drug showed statistically significantly higher expression of p21, p27 and activation of caspase 3 and lower expression of the proliferation marker Ki67 *vs* control vehicle tumors (Fig. 4D, E). Collectively, these findings demonstrate that the inhibition of neddylation prevents the tumorigenic potential of FP-RMS cells in vivo.

### MLN4924 enhances cell cycle arrest and apoptosis after irradiation in FP-RMS cells

**Fig. 5:**
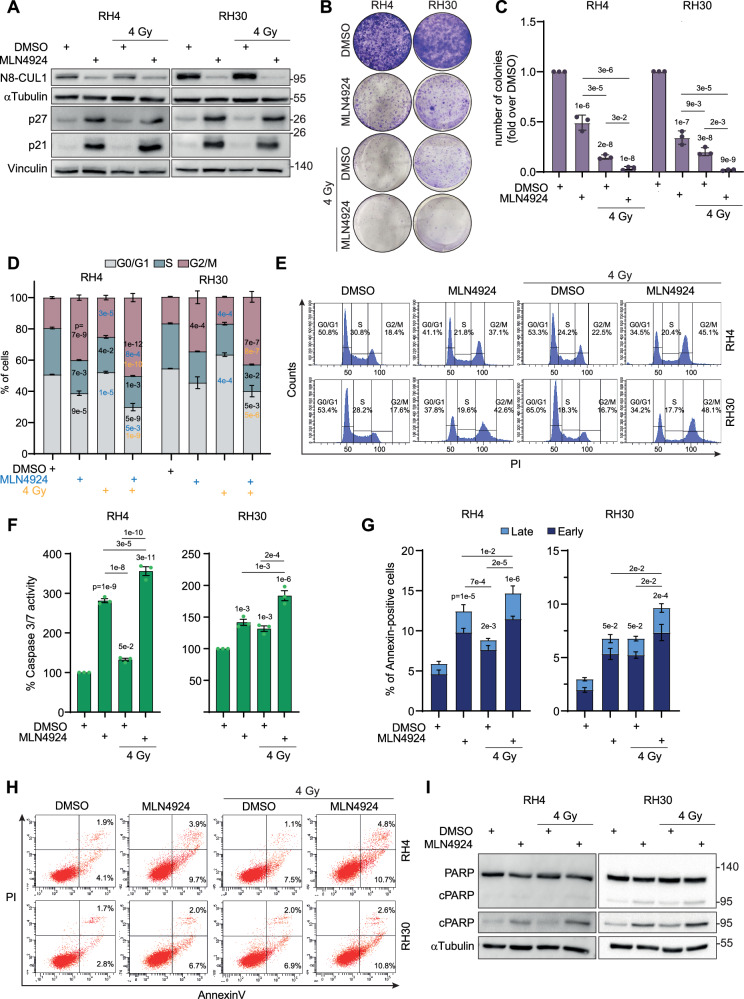
MLN4924 enhances cell cycle arrest and apoptosis after radiation in FP-RMS cells. **A** Representative western blot (*n* = 3 independent experiments) of the indicated proteins on RH4 and RH30 cells treated for 24 h with either vehicle (DMSO) or MLN4924 GI_50_, and then irradiated or not with a single dose of 4 Gy. Cells were assayed 6 h post-irradiation. Vinculin and αTubulin are the loading controls. **B** Representative images of RH4 and RH30 colonies treated as in (**A**). Colonies were stained with crystal violet 12 days post-cell seeding. **C** Histograms depict the number of colonies of RH4 and RH30 cells treated as in (**A**), calculated as a fold increase over vehicle (DMSO). Graph represents the mean ± SD (*n* = 3 independent experiments). One-way ANOVA. Exact *p*-values are reported in the figure. **D** RH4 and RH30 cells were treated for 24 h with either vehicle (DMSO) or MLN4924 GI_50_ and then irradiated or not with a single dose of 4 Gy and assayed after 24 h. Histograms depict the percentage of cells in G0/G1, S, and G2/M phases. Graph represents the mean ± SEM (*n* = 3 independent experiments). Two-way ANOVA. Exact *p*-values are reported in the figure. Black *p*-values: *vs* DMSO; green *p*-values: *vs* MLN4924; yellow *p*-values: *vs* 4 Gy. **E** Representative diagrams of cell cycle flow cytometry analysis on RH4 and RH30 cells treated as in (**D**). **F** Histograms depict the percentage of Caspase 3/7 activity of RH4 and RH30 cells treated as in (**D**), calculated as fold increase over vehicle (DMSO, 100%). Graph represents the mean ± SEM (*n* = 3 independent experiments). One-way ANOVA. Exact *p*-values are reported in the figure. **G** Histograms depict the percentage of Annexin-V positive/PI single- and double-positive RH4 and RH30 cells treated as in (**D**). Graph represents the mean ± SEM (*n* = 3 independent experiments). One-way ANOVA. Exact *p*-values are reported in the figure. **H** Representative plots of flow cytometry analysis of Annexin V/PI staining of RH4 and RH30 cells treated as in (**D**). **I** Representative western blot (*n* = 3 independent experiments) of the indicated proteins on RH4 and RH30 cells treated as in (**D**). αTubulin and Vinculin are the loading controls.

The DNA damage-inducing effects of MLN4924 prompted us to investigate whether this treatment boosts the response to irradiation, which is known to induce DSBs [25]. To this end, RH4 and RH30 cells were pre-treated with MLN4924 or vehicle for 24 h and, then, irradiated or not at 4 Gray (Gy), a dose which induces mild effects on FP-RMS cells [26], and assayed at the indicated time points. As expected, MLN4924 downregulated N8-CUL1 protein levels and upregulated p21 and p27 in both irradiated and not irradiated cells 6 h post-irradiation (Fig. 5A). Functionally, 24 h post-irradiation the drug significantly enhanced the irradiation-induced inhibition of colony formation by approximately 13- and 4-fold in RH4 and 21-and 12-fold in RH30 compared to MLN4924 and irradiation as single treatments, respectively (Fig. 5B, C).

As we already reported [26], 4 Gy of irradiation did not markedly change the cell cycle distribution of the two FP-RMS cell lines after 24 h. However, cells accumulated in the G2/M phase to a greater extent in response to the combination of MLN4924 and irradiation than to the drug alone (1.24- and 1.25-fold increase in RH4 and RH30 cells, respectively) and irradiation (2- and 2.5-fold increase in RH4 and RH30 cells, respectively) (Fig. 5D, E). In line, the percentage of cells in G1 phase was strongly reduced in the combination treatment *vs* MLN4924 (1.30- and 1.6-fold decrease in RH4 and RH30 cells, respectively) and irradiation (1.75- and 1.14-fold decrease in RH4 and RH30 cells, respectively) as single treatments.

This finding was associated with further induction of caspase 3/7 activity after MLN4924 and irradiation compared to vehicle and each treatment alone (1.3- and 2.7-fold in RH4 and 1.3- and 1.4-fold in RH30 *vs* MLN4924 and irradiation alone, respectively) (Fig. 5F). In accordance with this data, MLN4924-treated cells exhibited a significant increment of Annexin V-positive cells after irradiation (1.66- and 1.18-fold in RH4 and 1.42- and 1.41-fold in RH30 *vs* irradiation and MLN4924 alone, respectively) and increased levels of cleaved PARP *vs* single approaches (Fig. 5G–I). Collectively, these findings suggest that neddylation supports the cell survival of irradiated FP-RMS cells and its inhibition promotes G2/M cell cycle arrest and apoptosis of irradiated cells.

### MLN4924 increases irradiation-dependent DNA damage and DSBs in FP-RMS cells

**Fig. 6:**
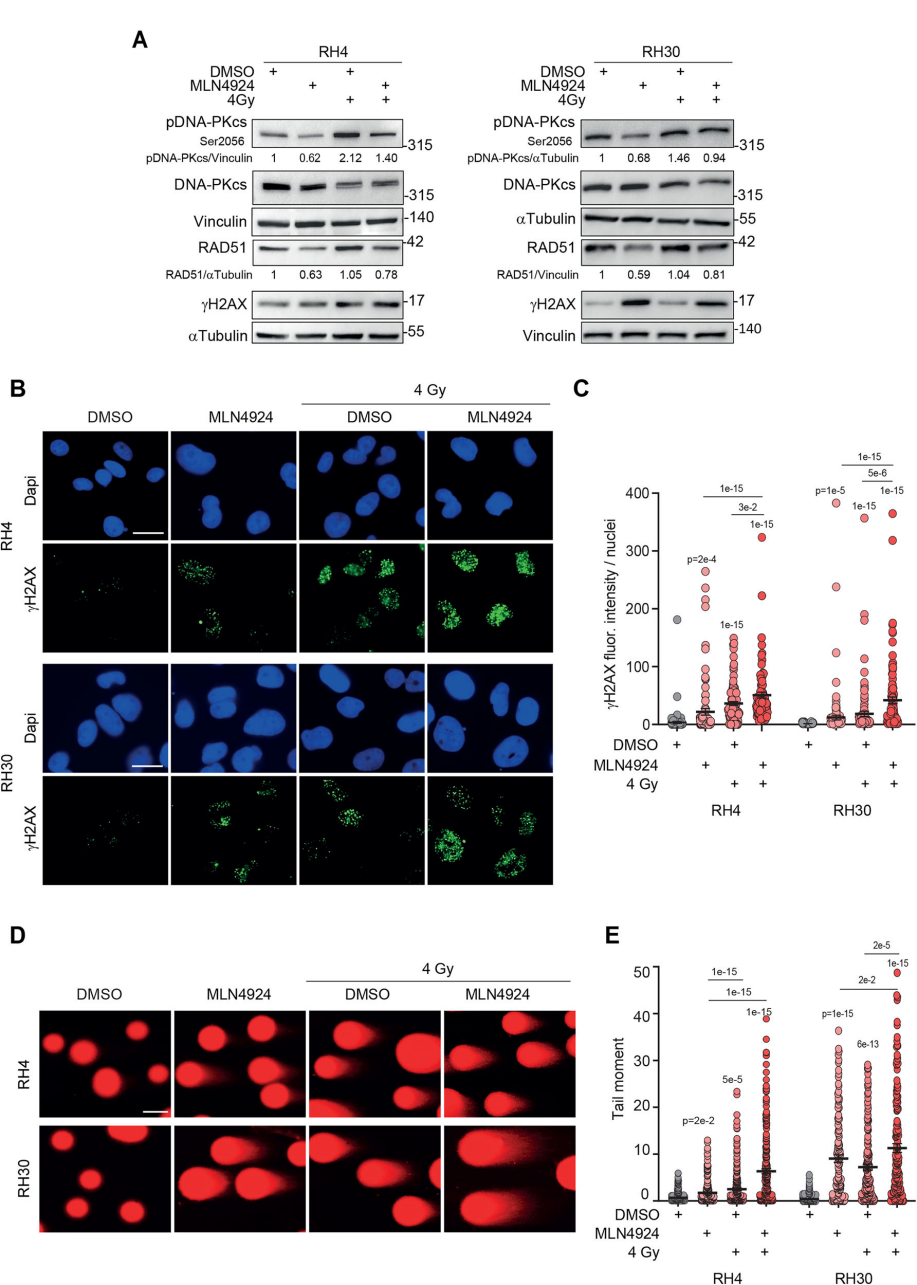
MLN4924 increases radiation-dependent DNA damage and DSBs and reduces anchorage-independent growth in FP-RMS cells. **A** Representative western blot (*n* = 3 independent experiments) of the indicated proteins on RH4 and RH30 cells treated for 24 h with either vehicle (DMSO) or MLN4924 GI_50_ and then irradiated or not with a single dose of 4 Gy and assayed 6 h post-irradiation. Vinculin and αTubulin are the loading controls. **B** Representative immunofluorescence images of γH2AX (green) on RH4 and RH30 cells treated as in (**A**). Dapi (blue) was the nuclear counterstain. Images were acquired with a fluorescence microscope equipped with a 60× oil immersion objective. Scale bar = 25 μm. **C** Scatter dot plots depict the intensity of γH2AX staining per nucleus on RH4 and RH30 cells treated as in (**A**). The graph represents the mean ± SEM. Kruskal-Wallis test. Exact *p*-values are reported in the figure. **D** Representative immunofluorescence images of the neutral Comet assay on RH4 and RH30 cells treated as in (**A**) and assayed 24 h post-irradiation. Images were acquired with a fluorescence microscope equipped with a 40× oil immersion objective. Scale bar = 50 μm. **E** Scatter dot plots represent the mean of the tail moment ± SEM. One-way ANOVA. Exact *p*-values are reported in the figure.

We next evaluated the extent of DNA damage induction by MLN4924 in irradiated cells. In both cell lines, treatment with the drug resulted in increased protein levels of γH2AX in irradiated cells compared to irradiation alone 6 h post-irradiation (Fig. 6A). MLN4924 also markedly impaired the irradiation-dependent increase of the protein levels of phosphorylated/activated DNA-PKcs, needed for DNA repair through the non-homologous end-joining (NHEJ) pathway, and reduced those of RAD51 in irradiated and not irradiated cells *vs* vehicle treated cells (Fig. 6A). MLN4924 and irradiation as single treatments induced 6- and 10-fold increase respectively in RH4 and 4- and 7-fold increase in RH30 in nuclear fluorescence intensity for γH2AX foci *vs* vehicle (Fig. 6B, C). Furthermore, fluorescence intensity of nuclear γH2AX foci in co-treated cells displayed 2.3- and 1.4- in RH4 and 3.8- and 2.4- in RH30 fold increase *vs* MLN4924 and irradiation, respectively (Fig. 6B, C). Accordingly, the drug enhanced the tail moment of irradiated cells by 3.7- and 2.5-fold in RH4 and 1.2- and 1.6-fold in RH30 24 h post-irradiation compared to irradiation and drug alone, indicating an increase in DSBs (Fig. 6D, E). Altogether, our results suggest that neddylation inhibition amplifies irradiation-induced double-strand DNA damage, possibly impairing DNA repair.

### MLN4924 in combination with irradiation inhibits anchorage-independent and spheroid growth in FP-RMS cells

**Fig. 7:**
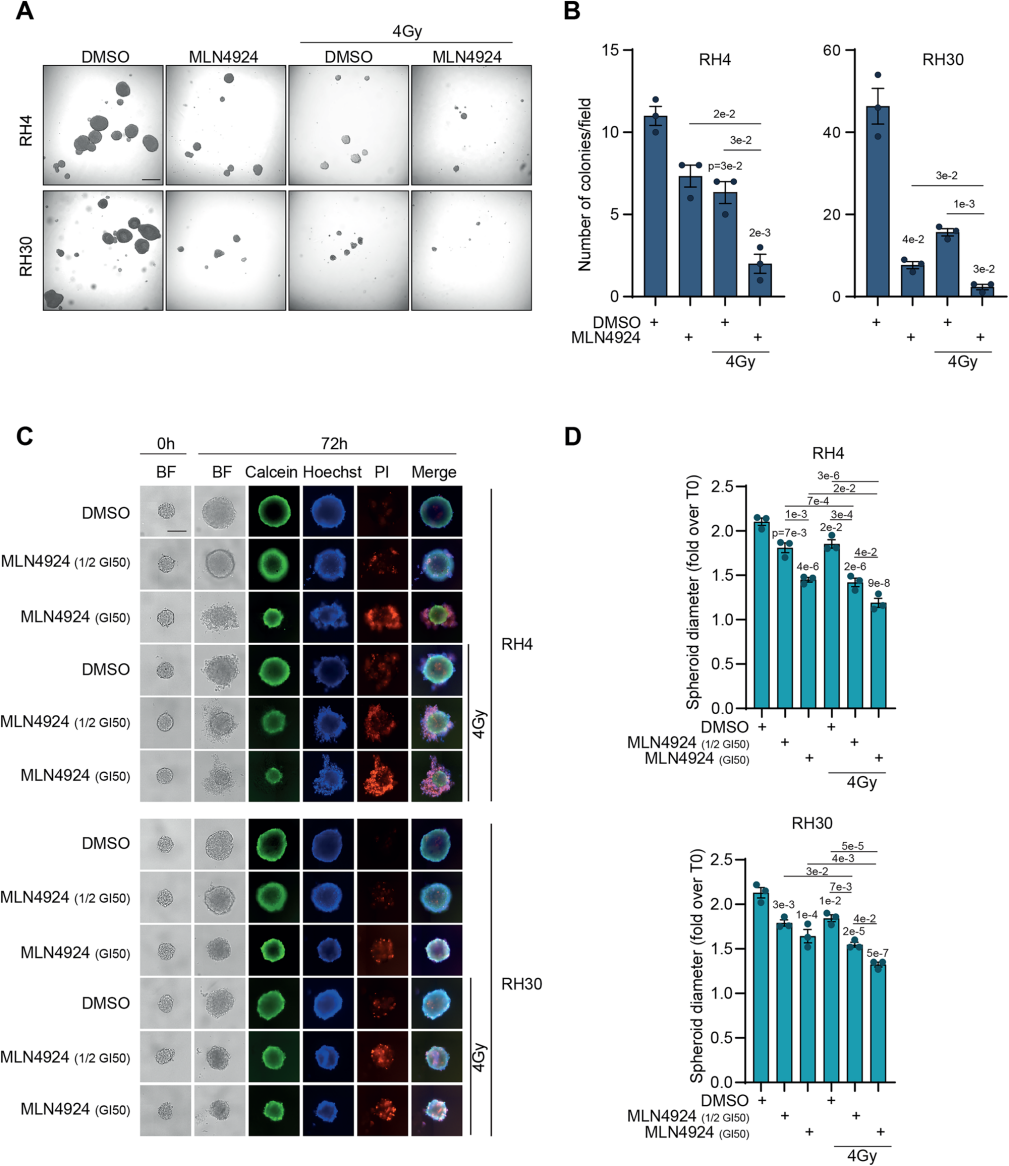
MLN4924 radiosensitizes FP-RMS cells growing as spheroids. **A** Representative image of the soft agar assay of RH4 and RH30 cells pretreated for 24 h with either vehicle (DMSO) or MLN4924 GI_50_, and then irradiated or not with a single dose of 4 Gy and grown for 2 weeks. Scale bar = 500 μm. **B** Histograms depict the number of colonies per field of RH4 and RH30 cells treated as in (**A**). Graph represents the mean ± SEM (*n* = 3 independent experiments). One-way ANOVA. Exact *p*-values are reported in the figure. **C** Representative images of RH4 and RH30 cells grown as single spheroids. FP-RMS spheroids were treated at T0 with either vehicle (DMSO) or MLN4924 (1/2 GI_50_ or GI_50_) and then irradiated or not with a single dose of 4 Gy and grown for 72 h. Spheroids were stained with Calcein (green), Hoechst (blue), and PI (red), and diameters were measured. Scale bars = 200 µm. **D** Histograms depict the diameter of RH4 and RH30 spheroids treated as in (**C**) and calculated as a fold increase over T0. Graphs depict mean ± SD (*n* = 3 independent experiments). One-way ANOVA: Exact *p*-values are reported in the figure.

To clarify the effects of MLN4924 on the tumorigenic potential of irradiated FP-RMS cells in vitro, we evaluated the ability of tumor cells to grow in the absence of anchorage, an ability closely related to carcinogenesis in vivo [27]. As shown in Fig. 7A, B, MLN4924 alone was able to reduce the number of cell colonies growing in soft agar by approximately 39% in RH4 and 84% in RH30 compared to vehicle-treated ones. The number of colonies was also decreased after irradiation by approximately 45% in RH4 and 66% in RH30 compared to vehicle-treated cells. Strikingly, pre-treatment with MLN4924 strongly impaired the ability of irradiated cells to form colonies in soft agar, further reducing their number by approximately 74% and 69% *vs* MLN4924 alone and approximately 70% and 85% *vs* irradiation alone, in RH4 and RH30 cells, respectively.

We, then, cultured the two FP-RMS cell lines in 3D as spheroids for 72 h in the presence of serum to mimic in vivo growth conditions and treated them with either MLN4924, irradiation, or their combination. MLN4924, as a single agent at either ½ GI_50_ or GI_50_ dose, reduced the growth of spheroids in a dose-dependent manner by approximately 14% and 31% in RH4 and about 16% and 23% in RH30 compared to vehicle (Fig. 7C, D). This decrease was associated with the appearance of a propidium iodide (PI)-positive population, indicating cell death, especially in GI_50_-treated spheroids. Irradiation alone hindered 3D growth by about 12% and 13% *vs* vehicle-treated RH4 and RH30 cells, respectively (Fig. 7C, D). The combinations of ½ GI_50_ and GI_50_ MLN4924 with irradiation further reduced spheroids’ growth by approximately 22% and 18% in RH4 and 14% and 20% in RH30 cells, compared to the drug alone, and by approximately 23% and 36% in RH4 and 16% and 28% in RH30 cells compared to the irradiation alone, respectively. Importantly, the PI-positive dead cells population appeared clearly more evident in the samples treated with the combinations compared to single treatments.

Finally, we evaluated the capacity of FP-RMS cells to form and grow as spheroids after treatment with the drug (GI_50_), irradiation, or in combination (Supplementary Fig. S3). Even in this case, although the size of 3D structures was reduced by approximately 15% and 30% in RH4 and 26% and 11% in RH30 cells with the drug and irradiation alone *vs* vehicle respectively, the combination of the treatments led to a further reduction of approximately 30% and 15% in RH4 and 22% and 36% in RH30 cells compared to MLN4924 and irradiation as single treatments, respectively. In summary, these findings suggest that neddylation is needed for the tumorigenic potential and resistance to irradiation in vitro of FP-RMS cells.

## Discussion

Here, we demonstrate that neddylation is a novel vulnerability in the high-risk FP-RMS subtype and pharmacological targeting of NAE activity, which drives the neddylation, hampers the tumorigenic properties of the cells in vitro and in vivo. Analyzing publicly available gene profiling data, we show that *NAE1* and *UBA3*, the regulatory and catalytic subunits of the NEDD8-activated enzymatic complex NAE, are overexpressed in RMS patients’ *vs* healthy muscle tissues. Moreover, RMS patients show one of the highest expressions of the two genes across different adult and pediatric tumors. The biological relevance of these data is highlighted by the effects of gene perturbation by CRISPR/Cas9 KO (https://depmap.org/portal/achilles), showing that FP-RMS cell lines are among the most dependent on *NAE1* and *UBA3* expression for survival compared to different adult and pediatric tumor cell lines. In keeping with this, FP-RMS cells show high sensitivity to pharmacological inhibition of NAE by MLN4924, which is being investigated in Phase I and II clinical trials in adult and pediatric cancers [15–18]. Thus, to clarify the functional impact of neddylation in FP-RMS cells, we evaluated the effects of pharmacological inhibition of NAE on the tumorigenic cell properties using MLN4924. Our results clearly indicate that neddylation (i) promotes G2/M cell cycle progression of two FP-RMS cell lines, (ii) protects them from Caspase- and PARP1-dependent programmed cell death and senescence, and (iii) supports the capacity of single tumor cells to survive over time growing into a colony, all abilities that were markedly hindered by MLN4924. Moreover, drug treatment was also able to prevent in vivo tumor growth of FP-RMS cell line xenografts, activating Caspase 3 and reducing the proliferation marker Ki67. The anti-proliferative/survival effects of the drug were consistent with the upregulation of p21 and p27 protein levels in vitro and in vivo. This last effect is partly due to p21 and p27 protein stabilization following the indirect inhibition of SKP2, the F-box subunit of the ubiquitin protein E3 ligase complex CRL1, as a result of CUL1 neddylation blockade [9, 10, 28], as we recently demonstrated in FN-RMS [11]. Upregulation of p27 had also been shown previously by the group of Charles Keller [28] in one of our target cell lines, RH30, after SKP2 silencing. However, an MLN4924-dependent inhibition of another E3 ligase, CRL4, could also additionally contribute to the p21 upregulation in our context, as reported in melanoma [29]. Interestingly, unlike the differentiation effects of SKP2-specific genetic depletion we reported in FN-RMS cells [11], MLN4924 inhibited the expression of MYOG, one of the major myogenic genes strictly needed for myogenic differentiation. This effect of the drug has already been reported by us in FN-RMS cells [11] and by others in myoblasts [30]. This finding is in line with the evidence that myogenesis is preceded by p21-induced cell cycle arrest in the G1 phase [31], while MLN4924 results in p21 increase followed by cell cycle blockade in G2/M.

Notably, *NAE1* silencing and, partly, *UBA3* silencing mirrored the effects of MLN4924, suggesting that the responses to the drug are mainly related to *NAE1* functional inhibition in FP-RMS. The higher effects of *NAE1* knockdown could be due to the concomitant downregulation of UBA3 levels in *NAE1* siRNA cells, while UBA3 did not seem to regulate NAE1 protein levels. This aspect should be investigated in future studies. However, we cannot exclude that other pathways are involved in the response to MLN4924. Indeed, MLN4924 has a large number of targets, including non-CRL proteins [32–35] and can have NAE1-independent effects [36]. The senescent phenotype induced by the NAE inhibitor could be related to p21 and p27 upregulation, which play crucial roles in senescence [37, 38]. Particularly, p21 has been associated with irreversible senescence in response to MLN4924 in colon cancer, glioblastoma [19], and melanoma cells [29]. We also demonstrate that neddylation inhibition by MLN4924 per se induces an accumulation of γH2AX, the key marker of DNA damage, and a downregulation of RAD51 levels, needed for DNA repair, and promotes the formation of DSBs still evident 48 h after treatment in FP-RMS cells. Thus, unresolved DSBs could explain the induction of apoptosis we observed at this time point in vitro and the activation of Caspase 3 in vivo. In fact, DSBs, if not resolved quickly, can lead to apoptotic cell death.

Our data on the induction of G2/M cell cycle arrest, senescence, and DNA damage by MLN4924 associated with tumor growth reduction in vivo are in agreement with a number of preclinical studies in other cancers [39–42]. A study on a panel of pediatric cancer cell lines, including RMS, performed 15 years ago by the Pediatric Preclinical Testing Program (PPTP) group, reported intermediate in vivo activity of MLN4924. The drug was tested in vivo on RH41 cells, derived from RH4–FP–RMS cells, and RH30 cells, showing varying responses [43]. Differences in experimental conditions, such as tumor size at treatment initiation and dosage regimen, may account for variations in xenograft behavior observed in that study compared to ours. Recently, promising results of the NCT03323034 Phase I clinical study on MLN4924 in combination with Irinotecan and Temozolomide in recurrent or refractory pediatric solid tumors, including rhabdomyosarcoma, have been reported [18]. They showed that the combination is well tolerated in children, even if, being a number of patients already heavily treated with Irinotecan and/or Temozolomide, the comparison with single drugs appeared difficult.

Targeted therapies with a single agent seem to have disappointing results due to drug resistance, and, therefore, our study could be considered as a proof-of-concept for combinatorial therapeutic approaches using MLN4924.

Given that FP-RMS cells are intrinsically radioresistant [44, 45], (ii) radiation is a first-line therapeutic approach in this tumor subtype for local control, and (iii) irradiation exerts its function by inducing DNA damage, we decided to verify whether combining MLN4924 with irradiation could promote radiosensitivity in these tumor cells in vitro. Our results demonstrate that the combination markedly enhances the effects observed after single treatments, amplifying G2/M cell cycle arrest, apoptosis, and DNA damage associated with DSBs.

This increased sensitivity to programmed cell death of MLN4924-treated irradiated cells could be related to the reduction of the NHEJ and HR components, DNA-PKcs phosphorylation, and RAD51, both boosted by irradiation, suggesting that DNA repair programs induced by irradiation [46, 47] are hindered by the drug. Finally, treatment with MLN4924 prevents irradiated FP-RMS cells from growing as spheroids in 3D and in an anchorage-independent manner, both tumorigenic properties reminiscent of in vivo growth.

While MLN4924 itself affects multiple cellular processes in FP-RMS, including proliferation, apoptosis, senescence, DNA damage, and cell cycle arrest, each of these contributes to the final detrimental effects, the higher induction of DNA damage could be the central and particularly relevant mechanism when MLN4924 is combined with ionizing radiation.

Substantial accumulation of DNA damage is in keeping with the increased levels of γH2AX and the higher induction of DSBs, which are the most lethal form of DNA damage. This suggests that MLN4924 compromises the cells’ ability to effectively repair radiation-induced DNA lesions.

Therefore, although processes like senescence and G2/M arrest may dominate under single-agent treatment, DNA damage accumulation and impaired repair mechanisms could be the major drivers of enhanced efficacy in the combination setting.

However, the high induction of γH2AX and the mild decrease in DNA-PKcs activation and RAD51 levels compared to single treatments suggest that other pathways, among which oxidative stress and ROS production can contribute to γH2AX phosphorylation [48], deserve future investigation. Altogether, these findings support the potential of MLN4924 as a radiosensitizer in FP-RMS and highlight DNA damage response inhibition as a key therapeutic mechanism.

Our findings are in keeping with a number of preclinical data showing that MLN4924 fosters radiosensitivity amplifying DNA damage and hindering the activation of DNA damage response, as reported for colon carcinoma [49], head and neck squamous cell carcinoma (HNSCC) [50] and pancreatic [51] and breast cancers [52].

Collectively, our results indicate that inhibiting neddylation could have detrimental effects on FP-RMS growth and could be combined with irradiation to boost radiosensitivity.

However, although we and others have shown that normal healthy cells are weakly affected by MLN4924 treatments, also at high doses, compared to tumor cells [11, 39, 53], it must be considered that MLN4924 inhibits the entire neddylation pathway, which is essential for many physiological processes [54], and moreover MLN4924 can have neddylation-independent effects [36]. Therefore, a great effort to develop inhibitors against additional targets of the neddylation pathway is ongoing to improve specificity and selectivity and reduce side effects [55, 56].

Therefore, deeper investigations are needed on the neddylation process, which should also be context-dependent and aimed at identifying combinatorial interventions to avoid detrimental effects. In summary, our results demonstrate that the expression of key components of the neddylation pathway is dysregulated in high-risk FP-RMS and its pharmacological inhibition hinders the malignant phenotype in vivo. Moreover, they suggest that inhibition of neddylation may be used to promote radiosensitivity in this RMS subtype.

## Materials and methods

### Bioinformatic analyses

Vulnerability data from CRISPR genome screening for *NAE1* knock-out were retrieved from DepMap (24Q2, https://depmap.org/portal/achilles/). MLN4924 drug sensitivity data were obtained from the drug sensitivity AUC (CTD^2^) dataset from DepMap (https://depmap.org/portal/). Gene expression profile from affymetrix data of *NAE1* and *UBA3* gene in skeletal muscle and RMS patients in Supplementary Fig. 1A, B was retrieved from R2 platform (https://hgserver1.amc.nl/cgi-bin/r2/main.cgi) using Asman dataset (GSE9103), Williamson datasets (E-TABM-1202) and Barr dataset (GSE66533), while whose reported in Supplementary Fig. 1C, D was retrieved from GSE66533, GSE14333, GSE108474, GSE111678, GSE14827, GSE16011, GSE26673, GSE31684, GSE32676, GSE32701, GSE34620, GSE39671, GSE42743, GSE43580, GSE64019, GSE64415, GSE7553, GSE7696, GSE87371, GSE9843, GSE2658, GSE16476, GSE9891, GSE9103, GSE2109, and GSE7307. All the data were plotted with GraphPad Prism 10.2.3 (403).

### Cell lines

Patient-derived fusion-positive PAX3-FOXO1 RMS cell lines RH30 and RH4 were provided by Peter J. Houghton (Greehey Children’s Cancer Research Institute, San Antonio, Texas, USA). Both cell lines were profiled for Short Tandem Repeats (STR) by BMR Genomics (Padova, Italy, http://www.bmr-genomics.it). Several aliquots of the first culture for each cell line were stored in liquid nitrogen at −80 °C for subsequent assays. Cells were maintained in culture for a maximum of 3 months. RH4 and RH30 cells were cultured in DMEM high-glucose (Invitrogen, Carlsbad, CA, USA) supplemented with 10% fetal bovine serum (FBS), 1% of an L-glutamine solution, and 1% of a penicillin–streptomycin solution. Cell lines were cultured at 37 °C in a humidified atmosphere of 5% CO_2_/95% air and regularly checked for mycoplasma contamination.

### Transient RNA interference

Cells were transfected with 75 nM final concentration of siRNAs against either human *NAE1* (NAE1.1: GCCAUGGAAUUCUUACAAGAA; NAE1.2: CCAAGCAGUAUUGAAGAUAUA; NAE1.3: CCAGGAGUAUCUAACUAUCAA), *UBA3* (UBA3.1: GAGCAAAUGUAUUGCUUCUUU; UBA3.2: CGACACUUUCUAUCGACAAUU; UBA3.3: CCUCUAUUGAAGAACGAACAA) or with a non-targeting siRNA as control (SIC001) (Sigma-Aldrich, St Louis, MO, USA) using Oligofectamine (Invitrogen, Carlsbad, CA), according to the manufacturer’s instruction. Twenty-four hours after the transfection, the medium was replaced with fresh growth medium supplemented with 10% FBS, 1% L-glutamine, and 1% penicillin–streptomycin. Cells were harvested 48 h after transfection.

### Drugs

MLN4924 (Pevonedistat, HY-70062) were purchased from MedChemExpress (Monmouth Junction, NJ, USA) and dissolved in accordance with the manufacturer’s instructions in DMSO at 10 mmol/L.

### Determination of the GI_50_ and cell proliferation assay

The Growth Inhibition (GI₅₀) values were determined as previously described in [11]. Briefly, 1.5 × 10^3^ RH4 and RH30 cells were seeded into 384-well plates containing complete growth medium. After 24 h, the cells were treated with decreasing concentrations of MLN4924 (9 µM–0.05 nM) or with DMSO. Cell confluence was measured using the Celigo Image Cytometer (Nexcelom Bioscience, Lawrence, MA, USA), and GI₅₀ values were calculated 72 h post-treatment using GraphPad™ Prism version 10.2.3.

For proliferation experiments, 1.5 × 10^3^ RH4 and RH30 cells were seeded into 384-well plates, and after 24 h (*t*₀), media containing DMSO or MLN4924 at the calculated GI₅₀ concentration were added. The percentage of cell confluence was quantified every 24 h under phase contrast using the Celigo Image Cytometer (Nexcelom Bioscience, Lawrence, MA, USA).

Forty-eight hours after siRNA transfection, RH4 and RH30 cell numbers were determined using the automated cell counter Countess^TM^ 3 (Thermo Fisher Scientific, Lafayette, CO, USA). Cell viability was assessed by Trypan blue staining using siSCR as a reference.

### In vitro irradiation and colony formation assay

The Radgil2, X-ray irradiator, was used to deliver radiation (IR). The cells were treated for 24 h with either MLN4924 or DMSO, and then irradiated or not with a single dose of 4 Gy, 6 h post-irradiation, RH4 and RH30 cell lines were counted and seeded in 6-well plates (4 × 10^3^/well) in complete growth medium [57]. Medium was refreshed every 2 days, and after 14 days, cells were fixed and stained with a solution of 20% methanol/0.25% crystal violet. Images were acquired by the iBright™ CL1500 Imaging System (Thermo Fisher Scientific, Lafayette, CO, USA). The number of colonies was counted. Triplicate assays were carried out in three independent experiments. For DNA damage and spheroid growth analyses, the cells were treated for 24 h with either MLN4924 or DMSO, and then irradiated or not with a single dose of 4 Gy.

### Protein extraction and Western blot

Whole-cell lysates were prepared by homogenizing cells in RIPA lysis buffer (50 mM Tris, pH 7.4, 150 mM NaCl, 1% Triton X-100, 1 mM EDTA, 1% sodium deoxycholate, 0.1% SDS) supplemented with protease inhibitor cocktail (Sigma-Aldrich, St. Louis, MO, USA), 1 mM NaF, 1 mM Na_3_VO_4_, and 1 mM PMSF. The lysates were incubated on ice for 30 min, followed by centrifugation at 12,000×*g* for 20 min at 4 °C.

The supernatants were collected, and protein concentration was determined using the BCA Protein Assay Kit (Pierce, Life Technologies, Carlsbad, CA, USA) according to the manufacturer’s instructions. Protein lysates were supplemented with reducing SDS sample buffer (200 mM Tris-HCl, pH 6.8, 40% glycerol, 20% β-mercaptoethanol, 4% sodium dodecyl sulfate, and bromophenol blue) and boiled for 8 min at 95 °C. Proteins were resolved on 6% or 12% SDS-PAGE gels and transferred to Hybond ECL membranes (Amersham, GE Healthcare BioScience Corporate, Piscataway, NJ, USA). Membranes were blocked for 1 h with 5% non-fat dried milk in Tris-buffered saline (TBS), then incubated overnight at 4 °C with the appropriate primary antibody. After washing in TBS, membranes were incubated with the corresponding HRP-conjugated secondary antibody, diluted 1:5000 in 5% non-fat dried milk in TBS, for 1 h at room temperature. Detection was performed using Western Blotting Substrate (Thermo Scientific™) or Western Lightning ECL Pro (PerkinElmer, Waltham, MA, USA). Antibodies against SKP2 (H-435, sc-7164, 1:500), DNA-PKcs (sc-390849), p27 (sc-1641) were obtained from Santa Cruz Biotechnology Inc., (Santa Cruz, CA, USA); Vinculin (hVIN-1, V9131, 1:5000) was purchased from Sigma-Aldrich (St. Louis, MO, USA); MYOG (F5D-c, 1:200) was from DSHB (University of Iowa, Iowa City, IA, USA); α-TUBULIN (NB100-680, 1:5000) was from Novus Biologicals (Littleton, CO, USA). Antibodies against APPBP1 (NAE1, ab187142, 1:1000) and UBA3 (ab124728, 1:1000) were obtained from Abcam (Cambridge, UK). Antibodies against Phospho-Histone H2A.X (Ser139) (9718), p21 (2947), Phospho-DNA-PKcs (68716), FOXO1 (28805), PARP (83732), RAD51 (8875), Nedd8 (19E3, 2754), and all secondary antibodies were sourced from Cell Signaling (Beverly, MA, USA). All antibodies were used according to the manufacturer’s protocols.

### Cell cycle

FP-RMS cells were harvested by trypsinization 48 h after treatment with MLN4924, with the last 24 h being post-irradiation, or 48 h after siRNA transfection. The cells were then washed in cold phosphate-buffered saline (PBS) and fixed overnight at 4 °C in a cold solution composed of 50% PBS-5% FBS and 50% acetone/methanol (1:4 v/v). After fixation, the cells were centrifuged for 5 min at 1200 rpm, and the pellet was stained with 50 μg/ml RNase (Sigma-Aldrich, St. Louis, MO, USA) and 50 μg/ml propidium iodide (PI) (ThermoFisher Scientific, Rockford, USA) for 30 min in the dark at room temperature. The acquisition and analysis of the cell cycle were performed using a FACSCanto II flow cytometer equipped with FACSDiva 6.1 CellQuest™ software (Becton Dickinson Instrument, San Jose, CA, USA).

### Senescence β-galactosidase staining assay

RH4 and RH30 cells were either transfected with siRNAs or treated with DMSO or MLN4924 for 48 h, followed by fixation with 4% PFA for 15 min. β-galactosidase staining was conducted using the Senescence β-Galactosidase Staining Kit (9860, Cell Signaling Technology, Inc., Danvers, MA, USA) according to the manufacturer’s instructions. Images were captured using a 20× objective lens of the Leica DMi8 microscope (Leica Microsystems, Mannheim, Germany).

### Determination of Caspase 3/7 activity

RH4 and RH30 cells were seeded into black, flat-bottom 96-well plates at a concentration of 5000 cells per well. To evaluate apoptosis, cells were treated with MLN4924 at the indicated doses in triplicate after a 24-h incubation period to allow cell adhesion. Caspase-3/7 activity was measured 24 h post-irradiation and 48 h after MLN4924-treatment using the Caspase-Glo-3/7 assay (G8090, Promega, Madison, WI, USA) according to the manufacturer’s instructions. The activity was then analyzed using the EnSpire Multimode Plate Reader (PerkinElmer, Waltham, MA, USA).

### Annexin V Determination

To quantify apoptosis, 1.1 × 10^6^ RH4 and RH30 cells were seeded into a P100 plate. After 24 h, cells were either treated with MLN4924 for 48 h, with the last 24 h being post-irradiation, or transfected with siRNAs for 48 h. Subsequently, cells were harvested and the cell suspension was then incubated with FITC-conjugated Annexin V and 7-Aminoactinomycin D (7-AAD) in binding buffer for 15 min in the dark, using the Annexin V apoptosis detection kit (BD Pharmingen, San Diego, CA, USA) according to the manufacturer’s recommendations. The cells were analyzed using a FACS Canto II flow cytometer equipped with FACSDiva 6.1 CellQuest software (Becton Dickinson Instrument, San Jose, CA, USA).

### Immunofluorescence

Two hundred of RH4 and RH30 cells were cultured onto a 24 × 24 coverslip, left attaching for 24 h, and then treated with MLN4924 for 48 h. In the combinatory treatment with IR cells were pretreated with the drug for 24 h and then irradiated with 4 Gy and fixed 6 h post IR in 4% paraformaldehyde (PFA)/PBS for 10 min at room temperature, permeabilized in 0.5% Triton X-100/PBS for 15 min, blocked with 3% BSA/PBS 20 min at room temperature and incubated overnight with mouse Anti-phospho-Histone H2A.X (Ser139) Antibody (clone JBW301 05-636, Sigma-Aldrich, EMD Millipore corp., USA) diluted 1:1000 in 1% BSA/0,1% Saponin/PBS. Antibody binding was revealed using species-specific secondary antibodies coupled to Alexa Fluor 488 (1:500 Invitrogen, A11029). Nuclei were visualized by counterstaining with DAPI. Images were acquired with a Leica DMi8 microscope at a 60× oil immersion objective (Leica Microsystems, Mannheim, Germany). The intensity average phospho-H2AX fluorescence of at least 90 nuclei, deriving from three independent experiments, was calculated using ImageJ software.

### Neutral comet assay

DNA double-strand breaks induction was assessed using the Comet assay (single-cell gel electrophoresis) under non-denaturing conditions, following the method outlined in [58]. In summary, after treatment with MLN4924 for 48 h, RH4 and RH30 cells were collected, and the pellets were suspended in PBS and kept on ice. The cell suspensions were mixed with 0.5% LMP agarose at 37 °C, and then spread onto the agarose-coated slides. The cells embedded in agarose were lysed by immersing the slides in a lysis solution (30 mM EDTA, 0.1% sodium dodecyl sulfate (SDS)), incubated at 4 °C for 45 min in the dark, and at the end rinsed in 1× Tris Borate EDTA (TBE) running buffer (Tris 90 mM; boric acid 90 mM; EDTA 4 mM). After an electrophoretic run (18 min in 1× TBE buffer at 1 V/cm), the slides were rinsed with distilled H_2_O and fixed in ice-cold methanol. DNA was stained with 0.1% GelRed and observed under a fluorescence microscope (Leica DMi8, Leica Microsystems, Mannheim, Germany) with a 20× objective lens, connected to a CCD camera for image capture. The amount of DNA DSB breaks was examined using the software CometScore 2.0 (RexHoover.com: CometScore) and expressed in tail moment values (tail length × fraction of total DNA in the tail). The data from tail moments were analyzed using Prism software. A minimum of 30 cells were analyzed for each experiment, and at least three independent experiments were carried out.

### Soft-agar colony formation assay

The capacity of RH4 and RH30 to form colonies in soft agar was assessed as previously described in [59]. Briefly, a total of 10^4^ RH4 and RH30 cells, pretreated with MLN4924 for 24 h in combination or not with IR 4 Gy were collected 6 h post irradiation and suspended in DMEM (10% FBS) with 0.35% Agar (50081 NuSieve GTG Agarose, Lonza, Walkersville, MD, USA). Cells were plated on a 0.7% Agar layer in DMEM (10% FBS) in 6 multi-well plates. Every 2 days, the medium was refreshed. After 14 days, colonies were counted by microscopic inspection, and images were acquired with a Leica DMi8 microscope (Leica Microsystems, Mannheim, Germany). Triplicate assays were carried out in three independent experiments.

### Spheroids generation and image acquisition

For the 3D tumor spheroid formation, 200 cells per cell line were seeded into 100 μL of complete growth medium in 96-well ultra-low attachment plates (#7007, CORNING, New York, USA) as described in ref. [60]. The diameters of the spheroids were measured after 72 h (*t*_0_) using a Celigo image cytometer (Nexcelom Bioscience, Lawrence, MA, USA). The spheroids were then treated with the specified concentration of MLN4924 and subjected to irradiation at 4 Gy or left unirradiated. Six days post-seeding, the diameters were measured again, and the 3D tumor spheroids were stained with Propidium Iodide (0.1 mg/mL final concentration), Calcein AM (1 mM final concentration), and Hoechst (1:10,000). Image acquisition and analysis were conducted using the Celigo image cytometer (Nexcelom Bioscience, Lawrence, MA, USA). The capacity of RH4 and RH30 to form 3D tumor spheroids, after MLN4924 pretreatment in combination or not with IR 4 Gy was assessed as previously described in [61].

### In vivo xenograft models

NOD.Cg-Prkdcscid Il2rgtm1Wjl/SzJ (NSG) 6–8-week-old female mice (Charles River (www.criver.com) were used for xenograft experiments. Mice were maintained under sterile conditions with a 12-h light/12-h dark cycle, at 18–23 °C and 40–60% humidity. RH4 and RH30 cells (3.5 × 10^6^) were subcutaneously injected into the right flank of the mice. Once the tumors became palpable, the animal groups were randomly assigned for further experimentation, and tumor size was measured, in a blinded manner, twice a week for 3 weeks. MLN4924/Pevonedistat, dissolved in DMSO and diluted in 0.9% NaCl containing 30% PEG300 and 5% Tween-80 (final DMSO concentration = 5%), was administered subcutaneously at a dose of 50 mg/kg, or vehicle (the same mixture without the drug) was given once daily, 6 days a week for three weeks [11]. Five mice per group (RH4 and RH30) were treated either with vehicle or MLN4924. Tumor volume was measured using calipers. All animal experiments were conducted in accordance with the European Communities Council Directive N. 2010/63/ EU, the Italian Ministry of Health guidelines (DL 26/2014) and national ethical requirements, and approved by the Italian Health Ministry for the Children’s Hospital Bambino Gesù/Plaisant animal facility of Castel Romano in Rome, Italy (protocol number 291/2023-PR).

### Immunohistochemistry

Immunohistochemistry was carried out on 2 µm-thick sections from formalin-fixed, paraffin-embedded tissue. After dewaxing and rehydrating the sections, heat-induced epitope retrieval was performed by boiling the slides in EDTA (pH 9) (Dako, Glostrup, Denmark). To block endogenous peroxidase activity, the sections were treated with 3% hydrogen peroxide, followed by a mouse-to-mouse blocking reagent to inhibit endogenous mouse immunoglobulins, and an additional blocking step with 5% BSA. The sections were then incubated overnight at +4 °C with the following antibodies, mouse monoclonal α-p27Kip1 (1:50; SX53G8.5, sc-53871, Santa Cruz), mouse monoclonal α-MYOG (1:100; F5D-c, DSHB), rabbit polyclonal α-Cleaved Caspase 3 (1:300; #9661, Cell Signaling Technology), rabbit polyclonal α-p21Cipl (1:50, 12D1, Cell Signaling Technology, Cat# 2947) and α-Ki67 (ready to use; IR626, Dako). Detection of the primary antibodies was performed using the appropriate secondary biotinylated antibodies and a peroxidase DAB kit, with or without counterstaining with Gill’s hematoxylin (Dako, Carpinteria, USA). Negative controls were processed in parallel, omitting the primary antibody. Histological images were acquired and analyzed in a blinded manner by a technician and a pathologist. Light microscopy imaging was conducted using a Nikon E600 microscope equipped with NIS Elements BR software, with a 20× objective lens. Analysis was performed with the ImageJ 1.53t software.

### Statistical analysis

*P*-values have been calculated with GraphPad Prism 10.2.3 using one or two-way ANOVA for multiple comparisons, Kruskal–Wallis or Mann–Whitney tests for non-parametric comparisons, and *t*-test for single comparison. Statistically significant *p*-values (*p*-value less than 0.05) were reported within the figures. The sample size was estimated considering no significant variation within each group of data. For the animal experiments, we relied on the principle of using the smallest sample size possible. We estimated the sample size in order to detect a difference in averages of 2 standard deviations at the 0.05 level of significance with an 80% power. No sample-size calculation was performed for the in vitro experiments. Three biological replicates were performed for each condition in order to account for a reasonable range of variability among samples.

## Supplementary information

Supplementary Figures

Full and Uncropped Western Blots

## Data Availability

The data that support the findings of this study are available from the corresponding authors upon reasonable request.
