# Supplementary material for: Neddylation inhibition induces DNA double-strand breaks, hampering tumor growth in vivo, and promotes radiosensitivity in PAX3–FOXO1 rhabdomyosarcoma
Source: Cell Death Discov. 2025 Nov 3;11:496. doi: 10.1038/s41420-025-02787-0 (PMC12583468; doi:10.1038/s41420-025-02787-0)
Supplement: Supplementary file 1 — Supplementary Figures [file 41420_2025_2787_MOESM1_ESM.doc]

**Supplemental information**

**Neddylation inhibition induces DNA double-strand breaks hampering tumor growth in vivo and promotes radiosensitivity in PAX3-FOXO1 rhabdomyosarcoma**

**Supplemental figures**

**Fig. S1. The NEDD8-activating enzyme (NAE1) is up-regulated in RMS patients.**

**A, B.** Box plot depicts NAE1 and UBA3 expression (array data) among normal skeletal muscle tissues (n=29, GSE6798), FN-RMS (n=56 E-TABM-1202; n=25 GSE66533) and FP-RMS (n=44 E-TABM-1202; and n=33 GSE66533) patients. **C, D.** Box plot depicting *NAE1* and *UBA3* expression (array data) across skeletal muscle, rhabdomyosarcoma, mixed normal tissues and other human tumors. Box plots represent 25th to 75th quartiles, black bar depicts the median and whiskers go from the minimum value to maximum value. One-way ANOVA. Exact p-values are reported in the figure. One-way ANOVA Exact p-values are reported in the figure.


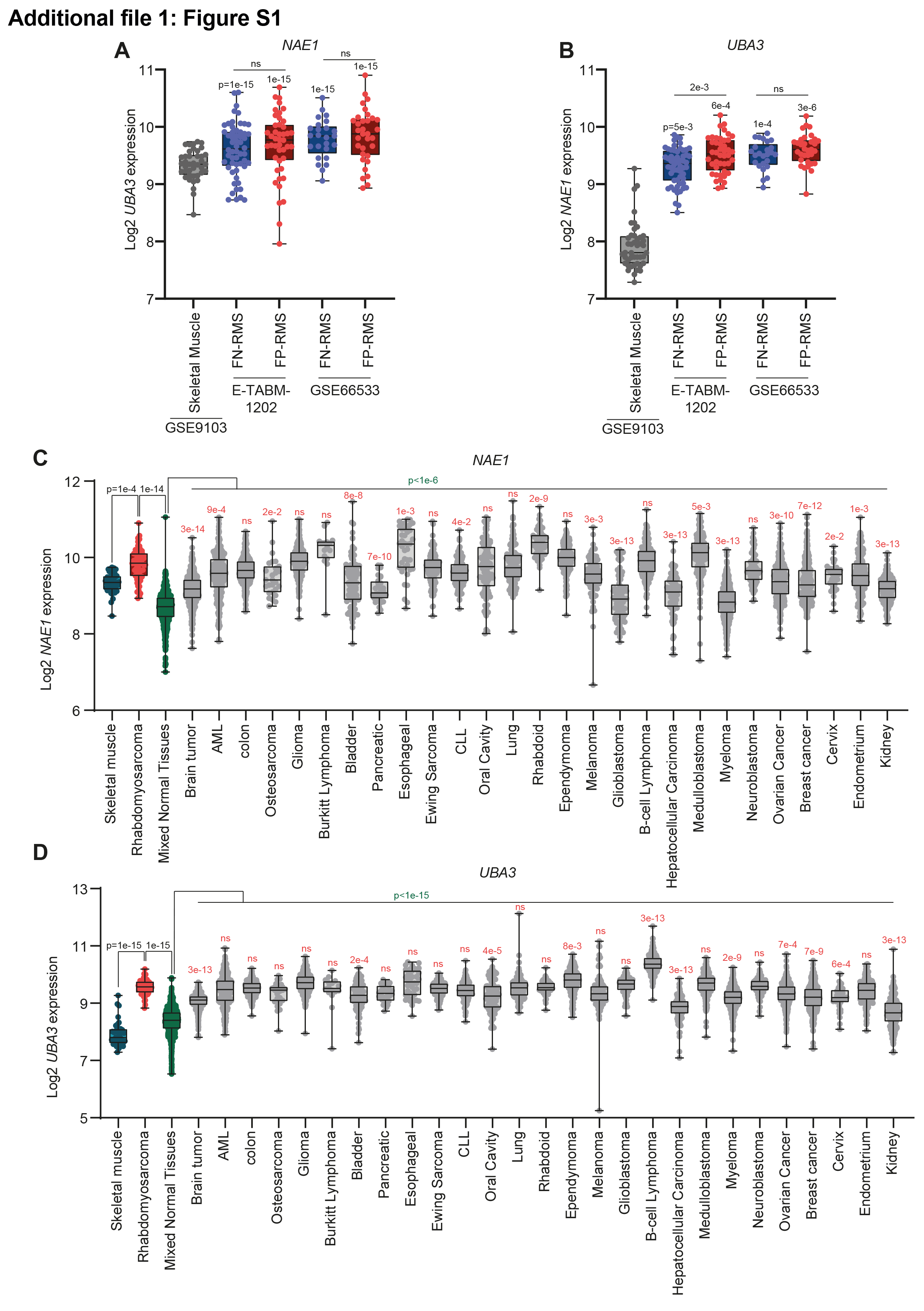


**Fig. S2. NAE3 and UBA1 knock down impairs cell proliferation and survival in FP-RMS cells.**

**A.** Western blot of the indicated proteins on RH30 cells transfected with SCR, NAE1.1, NAE1.2, NAE1.3, UBA3.1 UBA3.2, UBA3.3 siRNAs 48h post-transfection. αTubulin is the loading control. **B.** Cell count of RH4 and RH30 cells transfected with SCR, NAE1.2, UBA3.2 siRNAs 48h post-transfection. Graph represents the mean ± SD (n=3 independent experiments). One-way ANOVA. Exact p-values are reported in the figure. **C.** Western blot of the indicated proteins on RH4 and RH30 cells transfected as in (**B**). αTubulin and Vinculin are the loading controls. **D.** Histograms depict the percentage of RH4 and RH30 cells transfected as in (**B**) in G0/G1, S, and G2/M phases. Graph represents the mean ± SEM (n=3 independent experiments). Two-way ANOVA. Only the exact p-values relative to siScr are reported in the figure. **E.** Representative pictures of β-Galactosidase staining of RH4 and RH30 cells transfected as in (**B**). Scale bar = 100 µm **F.** Histograms depict the percentage of senescent cells per field (n=2 independent experiments). **G.** Histograms depict the percentage of Annexin-V positive/PI single- and double-positive RH4 and RH30 cells transfected as in (**B**). Graph represents the mean ± SEM (n=3 independent experiments). Two-tailed t-test. Only the exact p-values relative to siScr are reported in the figure.


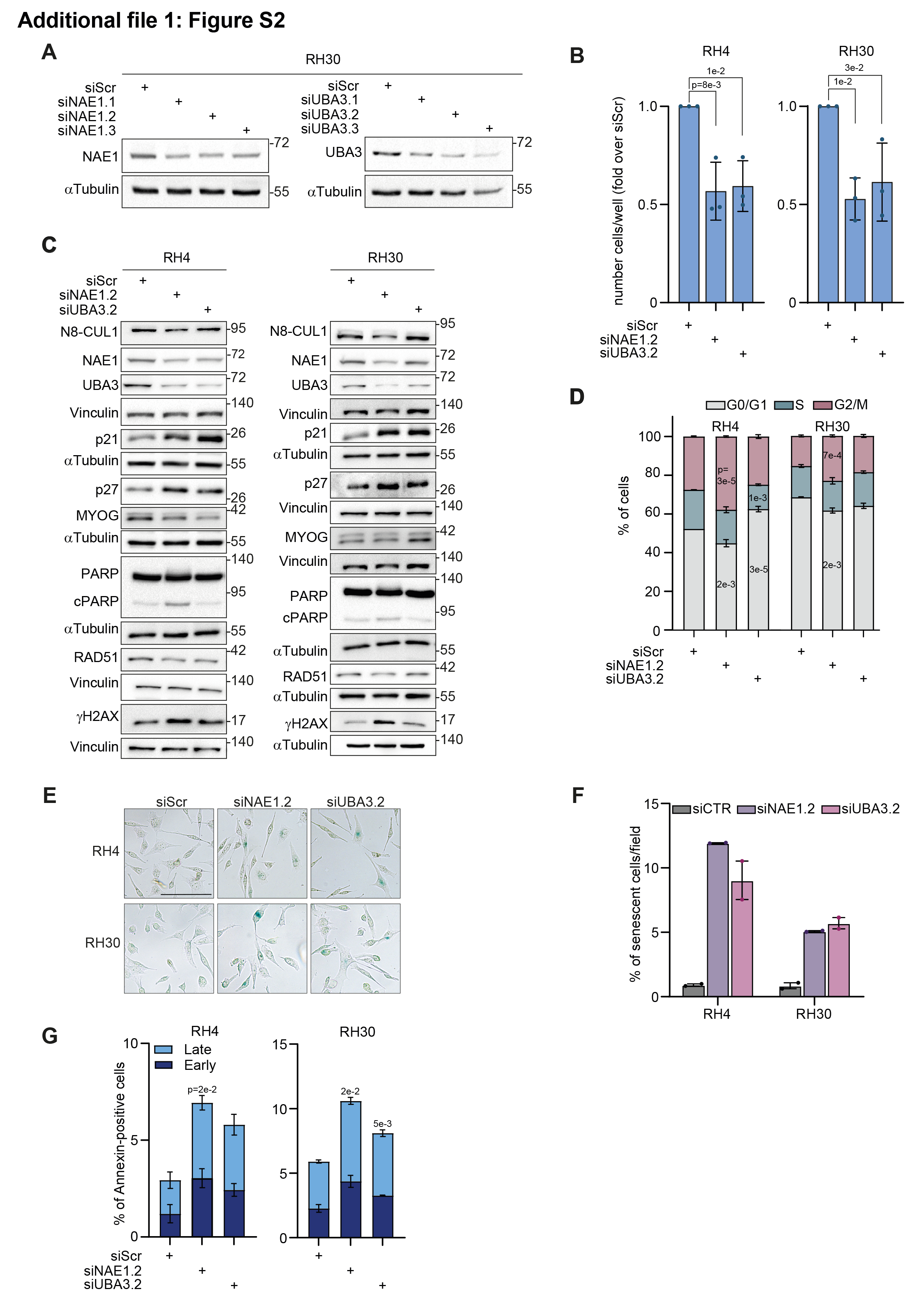


**Fig. S3**. **MLN4924 radiosensitizes FP-RMS cells growing as spheroids.**


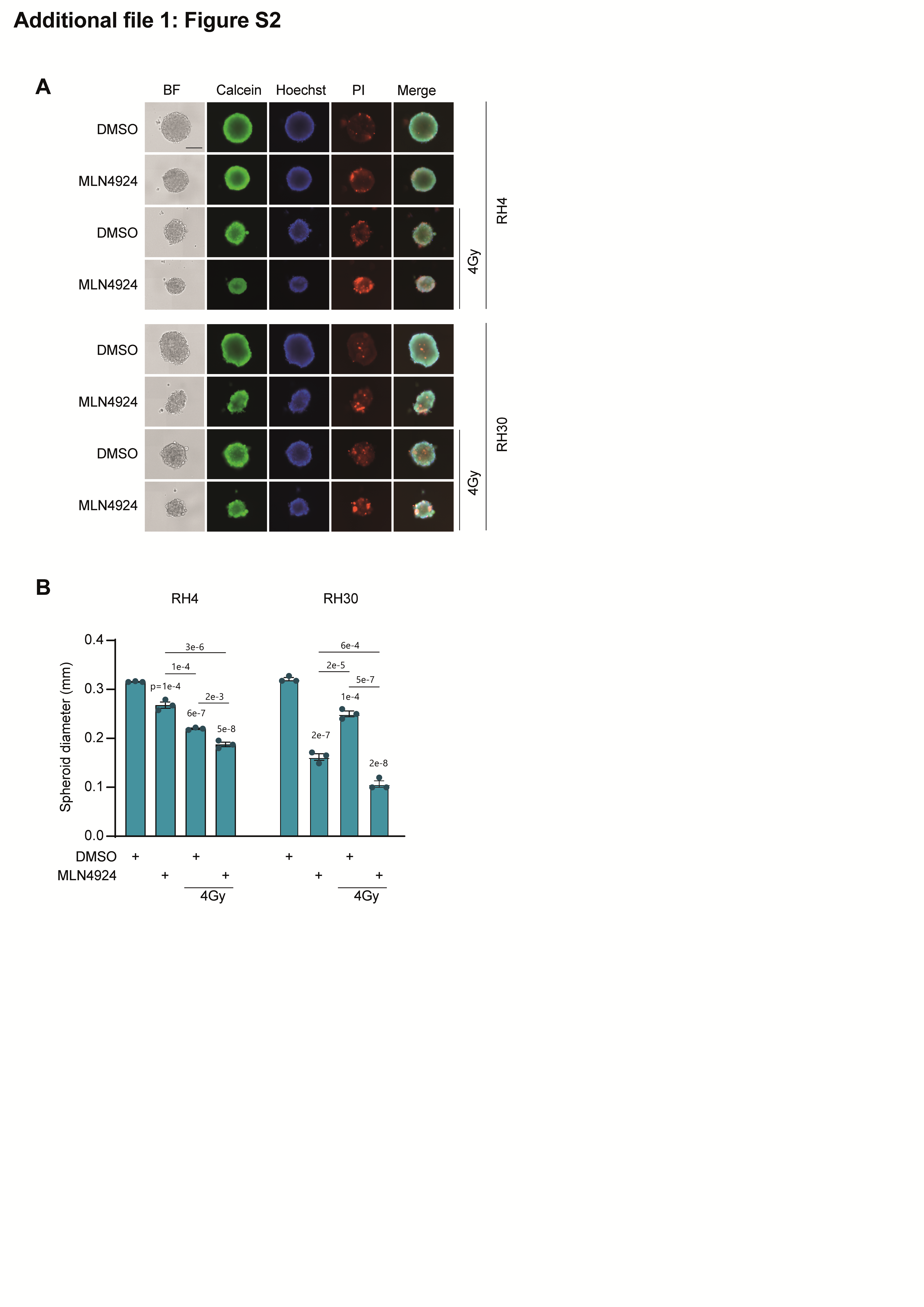
**A.** Representative images of RH4 and RH30 cells treated with either vehicle (DMSO) or MLN4924 GI50 and then irradiated or not with a single dose of 4Gy and grown as spheroid for 6 days. Spheroids were stained with Calcein (green), Hoechst (blue) and PI (red) and diameters were measured. Scale bars = 200 µm. **B.** Histograms depict the diameter of RH4 and RH30 spheroids treated as in (A). Graphs depict mean ± SD (n=3 independent experiments). One-way ANOVA. Exact p-values are reported in the figure.
