# Supplementary material for: Neddylation inhibition induces DNA double-strand breaks, hampering tumor growth in vivo, and promotes radiosensitivity in PAX3–FOXO1 rhabdomyosarcoma
Source: Cell Death Discov. 2025 Nov 3;11:496. doi: 10.1038/s41420-025-02787-0 (PMC12583468; doi:10.1038/s41420-025-02787-0)

Figure 2

C

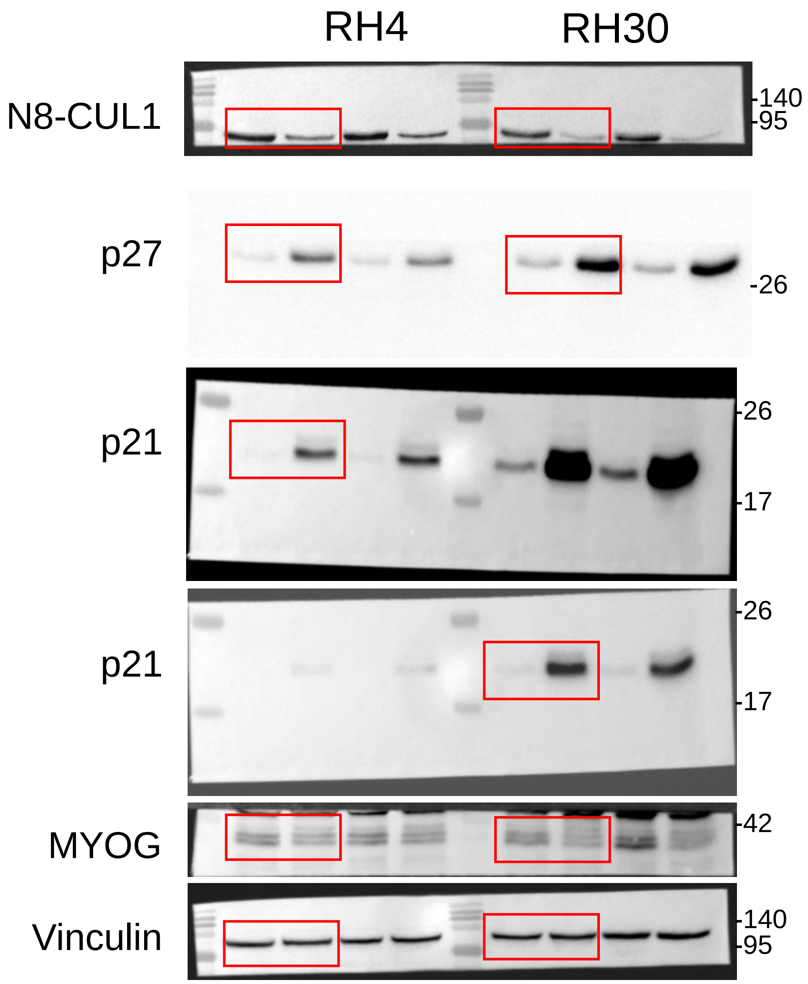

RH4
RH30
N8-CUL1
-140
-95
p27
-26
p21
-26
-17
p21
-26
-17
MYOG
-42
Vinculin
-140
-95

Figure 3

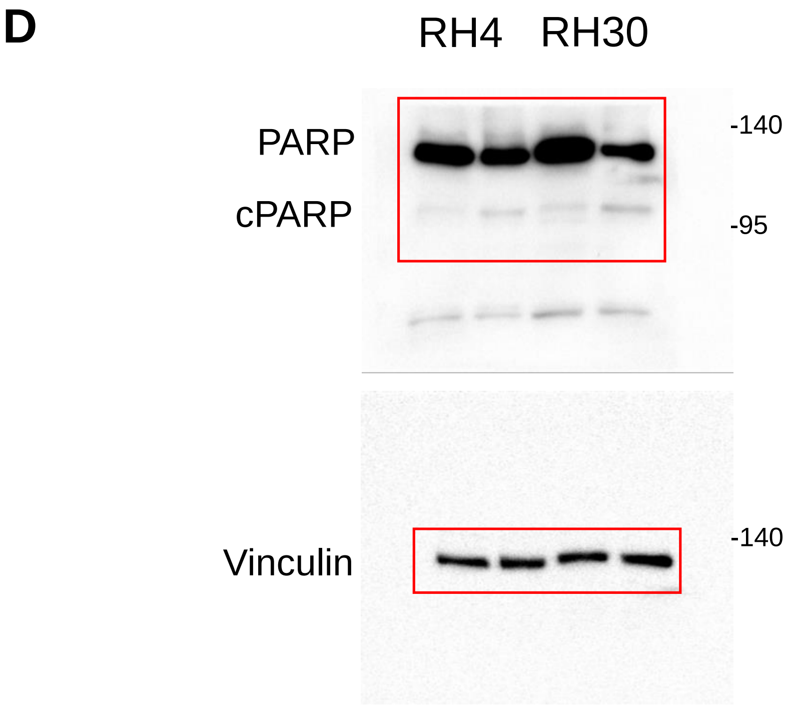
D
RH4
RH30
PARP
cPARP
-140
-95
Vinculin
-140

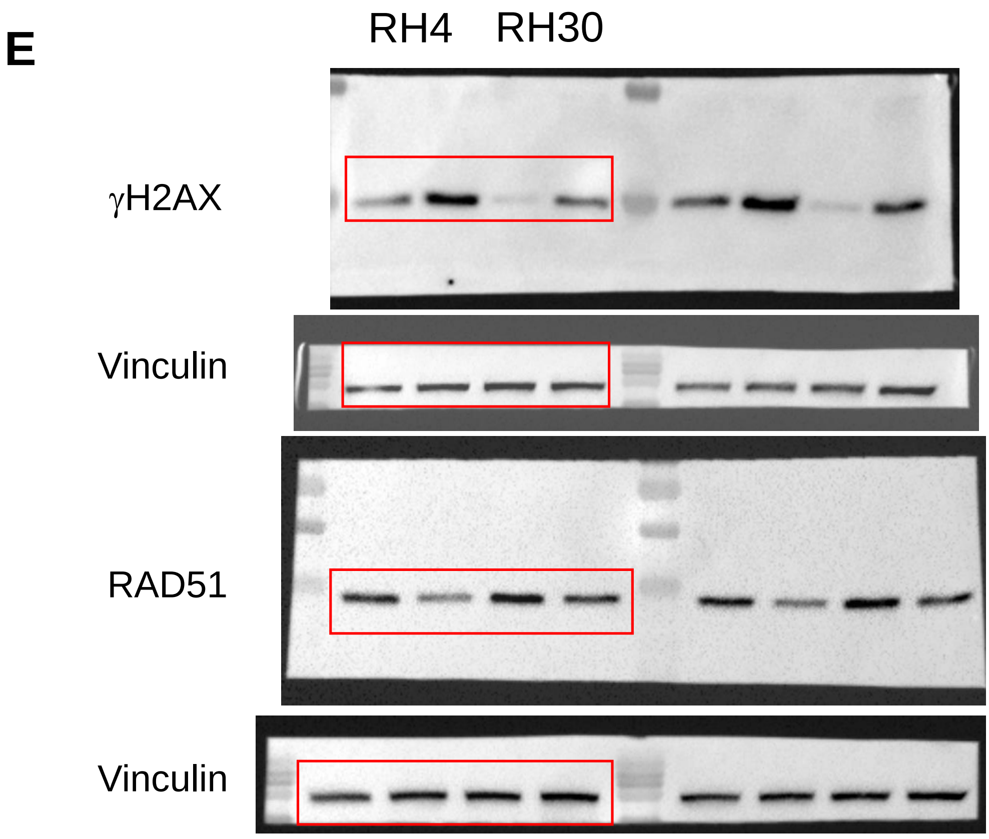
E
RH4
RH30
γH2AX
Vinculin
RAD51
Vinculin

Figure 5

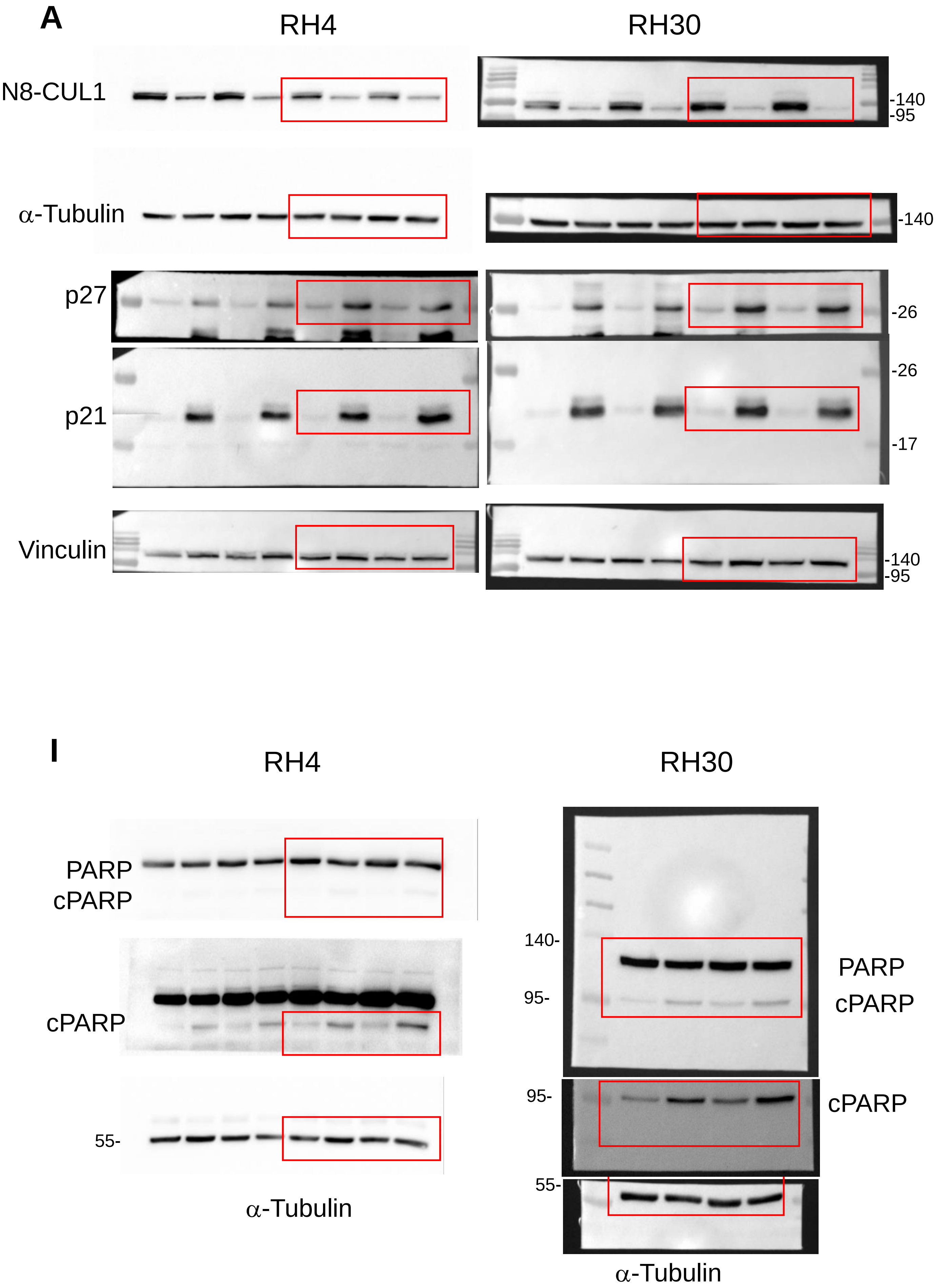

A
RH4
RH30
N8-CUL1
α-Tubulin
p27
p21
Vinculin
-140
-95
-140
-26
-26
-17
-140
-95
I
RH4
RH30
PARP
cPARP
cPARP
55-
α-Tubulin
140-
95-
PARP
cPARP
95-
cPARP
55-
α-Tubulin

Figure 6

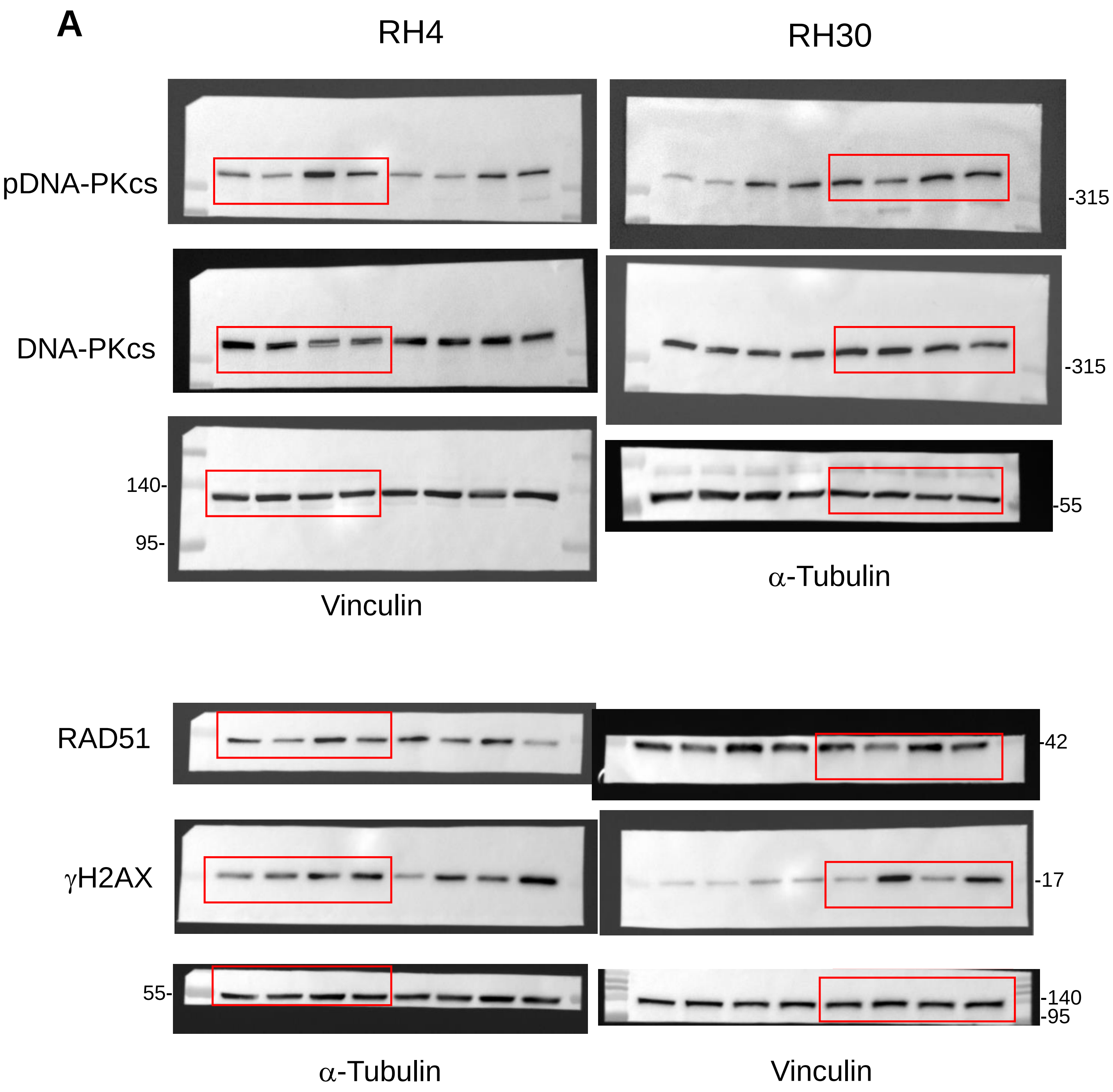
A
RH4
RH30
pDNA-PKcs
-315
DNA-PKcs
-315
140-
95-
-55
Vinculin
α-Tubulin
RAD51
-42
γH2AX
-17
55-
-140
-95
α-Tubulin
Vinculin

Additional file 1: Figure S2

A

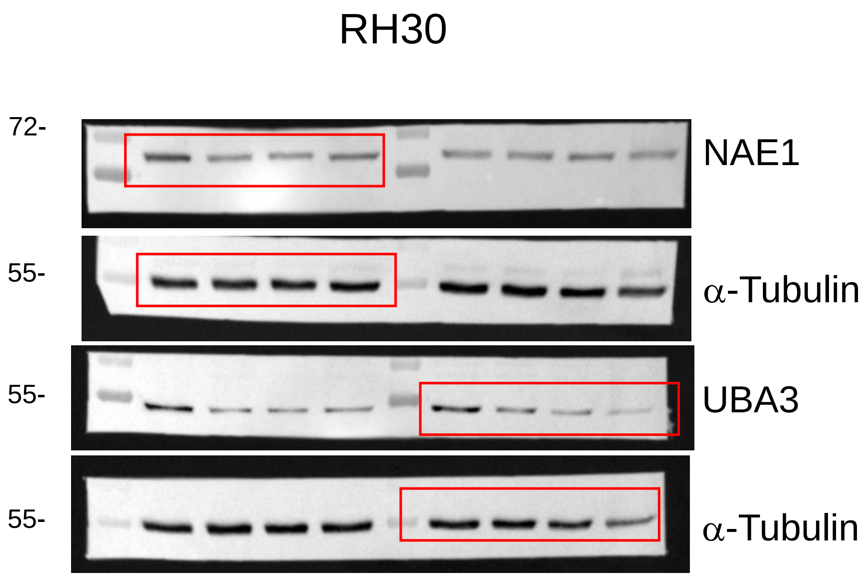

Additional file 1: Figure S2

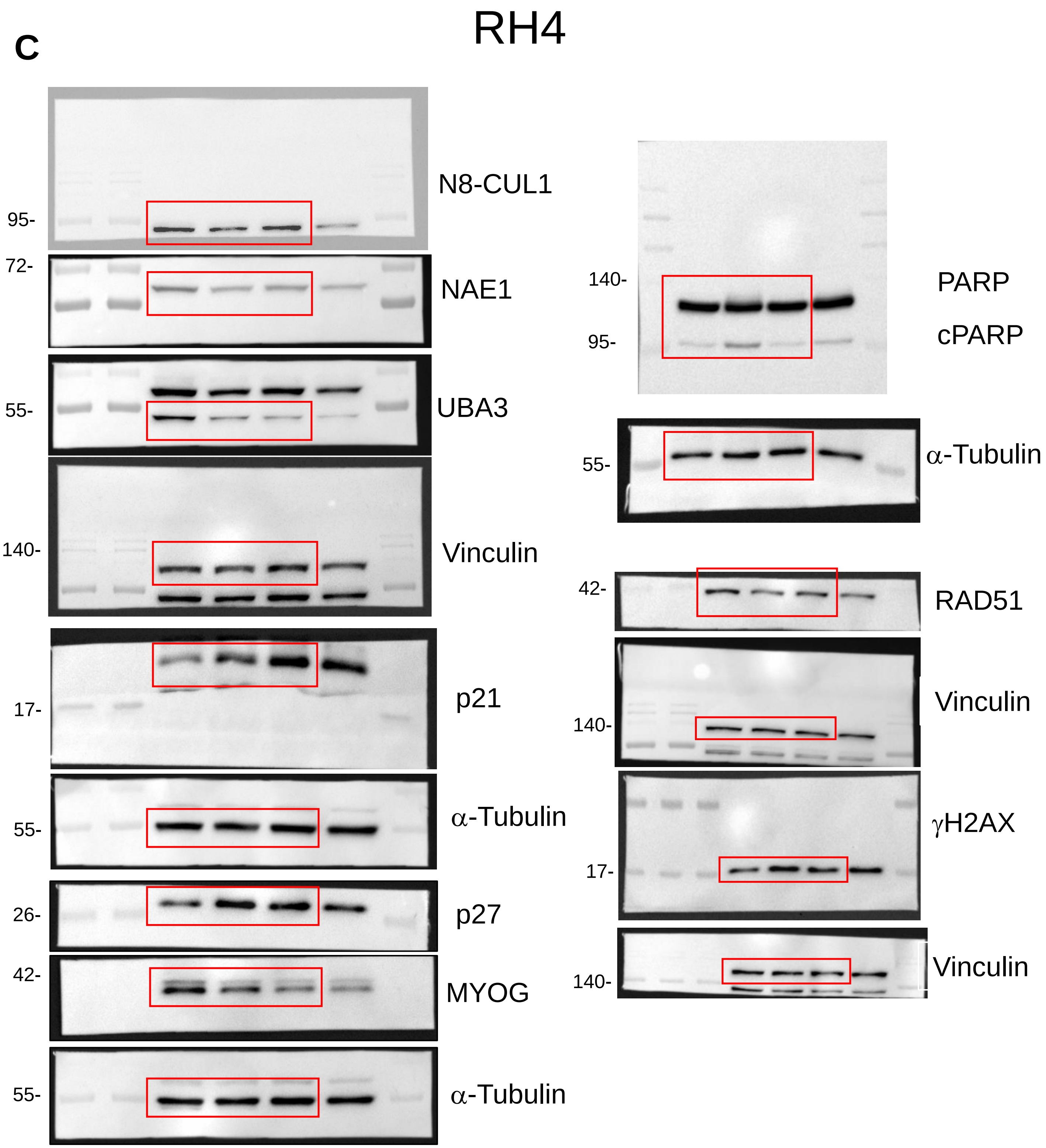

Additional file 1: Figure S2

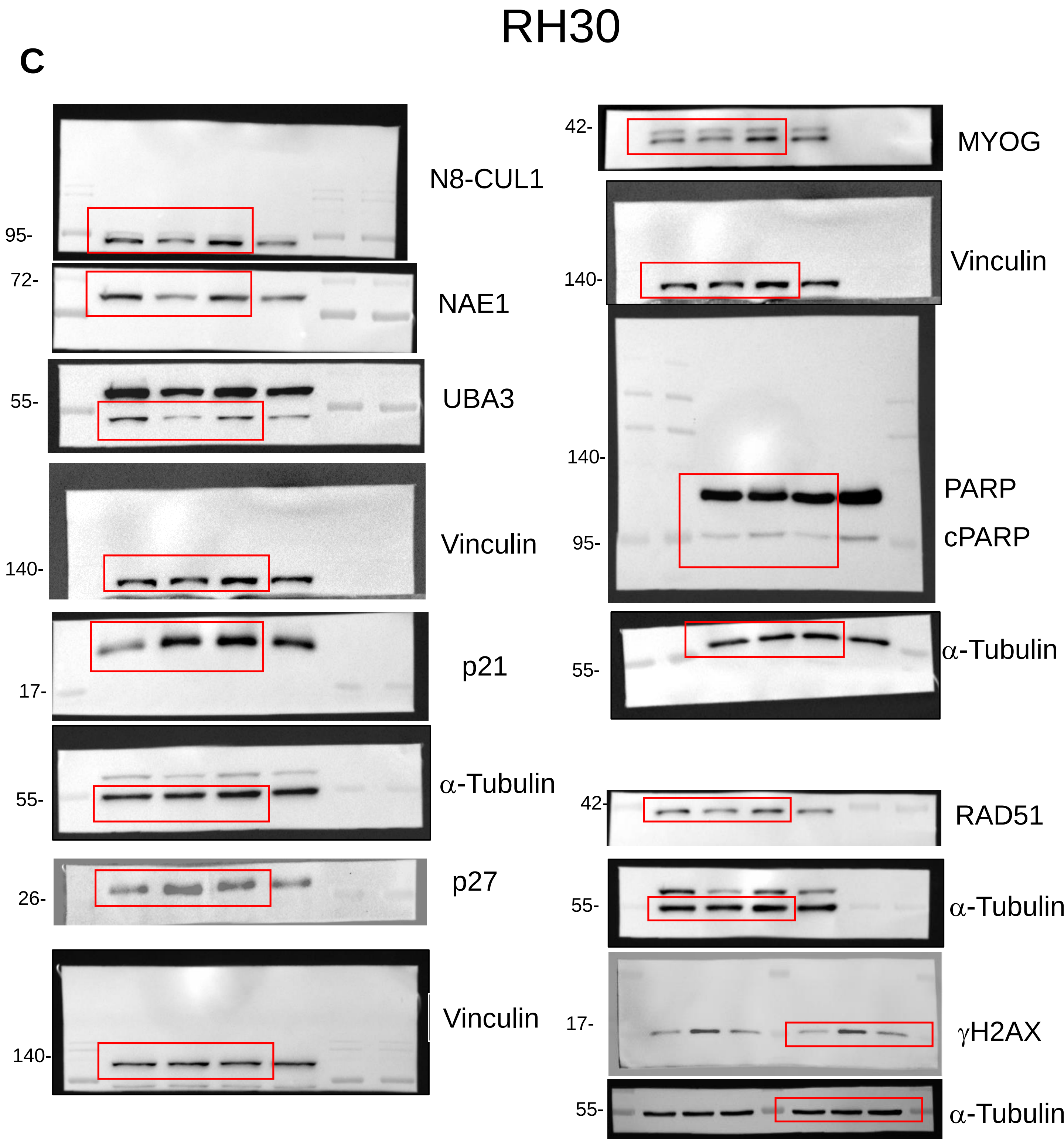

Supplement: Supplementary file 2 — Full and Uncropped Western Blots [file 41420_2025_2787_MOESM2_ESM.pdf]
